# Lumbar Acceleration Gait Estimation: “Step-by-Step” Algorithm Updates and Improvements

**DOI:** 10.2196/72831

**Published:** 2025-12-12

**Authors:** Lukas Adamowicz, Wenyi Lin, F Isik Karahanoglu, Xuemei Cai, Mar Santamaria, Charmaine Demanuele, Junrui Di

**Affiliations:** 1 AI/ML Quantitative Digital Sciences Pfizer Research and Development Pfizer Cambridge, MA United States; 2 Biomeasures, Endpoints, and Study Technologies Pfizer Research and Development Pfizer Cambridge, MA United States

**Keywords:** gait, accelerometer, IMU, walking, walking speed

## Abstract

**Background:**

Digital health technologies, such as accelerometry, offer low participant burden and provide quantitative metrics with ease of deployment, making them increasingly popular for gait monitoring. Remote gait monitoring delivers quantifiable, continuous health measures over extended periods, surpassing the limited insights from single clinic or laboratory visits and offering a more comprehensive health perspective. Numerous gait algorithm implementations, inspired by prior research, aim to standardize these metrics across devices. The SciKit Digital Health (SKDH) package exemplifies this as a device-agnostic framework.

**Objective:**

This study introduces a series of literature-informed enhancements to the SKDH gait algorithm, improving its performance against reference standards and reducing the need for manual parameter adjustments across diverse populations.

**Methods:**

A block-wise refinement process was undertaken, examining each algorithmic component for potential enhancements and evaluating their cumulative impact on the complete gait algorithm and the metrics generated.

**Results:**

Using data from healthy adult and pediatric participants, the novel gait event estimation method reduced the mean absolute error by more than 50% compared with its predecessor. Following the updates, the intraclass correlation values for final gait metric concordance with the in-laboratory reference improved markedly, from 0.50-0.74 to 0.81-0.90. Additionally, the systematic bias observed in the previous version’s gait speed estimation was rectified, narrowing the difference from the reference from 0.065-0.230 to 0.00-0.03 m/s.

**Conclusions:**

The findings from this study provide robust evidence supporting the validity of the enhancements made to the gait algorithm. They demonstrate that a single lumbar accelerometer can capture gait characteristics with high accuracy and reliability across various speeds and age groups.

## Introduction

Gait monitoring via digital health technologies, such as wearable inertial sensors, continues to grow in prevalence and acceptance. Especially given the ability of these digital health technologies to enable remote and continuous monitoring, these quantitative data have provided key insights for clinicians, including the ability to monitor or diagnose a variety of diseases and populations [1]. This increased acceptance is highlighted by the recent approval of the 95th percentile of gait speed as a primary end point in Duchenne Muscular Dystrophy trials [2]. Gait has also been referred to as a “functional sixth vital sign” [3,4], and, along with previous research showing relationships with all-cause mortality, disability status, age, and more [5-15], highlights the importance of continuous gait monitoring. With patient centricity as the main objective, remote and continuous gait monitoring provides opportunities for trial sponsors to consider decentralized clinical trials, which can reduce patient burden and increase patient diversity [16].

Researchers have developed algorithms to extract spatial and temporal gait metrics using data collected by sensors at various body locations, such as the wrist, hip, and lower trunk, which has enhanced the understanding of human locomotion dynamics [17-22].

Beyond the lumbar and wrist, numerous other sensor placements have been investigated. Foot- and shank-mounted inertial sensors have long been regarded as reference-standard placements, as they directly capture ground contact events and enable accurate stride length and speed estimation [22,23]. However, their use in large-scale or decentralized studies is limited by setup burden, footwear dependence, and reduced patient compliance compared with lumbar or wrist-worn devices. More recently, innovative placements such as head- and ear-worn sensors have been explored, leveraging hearing aids, earbuds, or headbands to unobtrusively capture gait dynamics [24-28]. These approaches demonstrate the diversity of wearable configurations under study, though many face challenges in patient compliance, real-world wearability, or algorithm generalizability.

To increase overall compliance and reduce participant burden, it is of great interest to use a single sensor to monitor bilateral gait. The lumbar location is the most popular choice, though it has its drawbacks. Namely, accuracy can sometimes suffer due to its distal location relative to the feet and ground contact points. This is especially true for spatial metrics, which rely on modeling steps as pendulum swings [22,29], as opposed to direct integration (which has its own numerical problems) or the location and trajectory estimation methods typically used for sensors placed on the feet or legs. This lower accuracy is generally the result of a relatively constant bias in spatial metric estimation, such as stride length and gait speed [7,30,31], even when algorithm precision remains high. Some previous works have addressed this drawback with personalized constants requiring calibration [32]; however, this potentially adds significant participant burden and limits the ability to conduct fully remote trials. Other remedies include using a relation between step frequency and step length [33], machine/deep learning approaches, or employing different sensor locations [34]. However, these may suffer from a lack of generalizability, as nonphysical models or relationships were employed in the estimation procedure. Therefore, it is of interest to address these drawbacks within the current physical inverted pendulum model and improve accuracy.

While the lumbar site has technical limitations due to reliance on pendulum modeling for spatial metrics, it also offers practical advantages. Compared with placements such as the feet, chest, or head, the lumbar sensor is generally easier for participants to wear consistently, less intrusive in daily activities, and less affected by soft tissue or motion artifacts. Previous work has highlighted the lumbar site as a practical compromise between accuracy and wearability, supporting its use in both older adults and patient populations where long-term compliance is critical [31,35]. These attributes, coupled with its ability to capture bilateral gait from a single device, make the lumbar location particularly appealing for large-scale monitoring and decentralized trial settings, thereby reinforcing the rationale for optimizing algorithms at this site.

While several algorithms have been developed to estimate gait parameters from sensors placed at various body locations, the options for a comprehensive solution that can be implemented in clinical trials remain limited. One such option is our open-source SciKit Digital Health (SKDH) [36], a Python package that includes various digital health algorithms (eg, sleep, physical activity, gait), data ingestion tools, and preprocessing methods. The gait module in SKDH, that is, SKDH-gait, evolved from GaitPy, a stand-alone open-source package [20] that provided an interface for estimating gait metrics within a fixed framework. While the majority of the gait implementation remained the same, additional options were added, such as a dynamic relation between step frequency and wavelet scale [37], which has already improved overall accuracy. Validation results for both the original GaitPy [7] and the SKDH-gait implementation [30,31] have been previously published. However, a direct comparison between the 2 versions has never been officially reported. Another issue with these implementations is that a population-specific parameter specification is required, depending on factors such as age, which can create an additional operational burden. Other gait algorithms include the recently published Mobilise-D gait algorithm [38,39] and the gaitmap project [40]. While the gaitmap project focuses primarily on foot-placed devices—requiring 2 sensors for bilateral assessment in addition to the drawbacks noted above—the Mobilise-D gait algorithm utilizes a single lumbar device and has been released as an open-source package on GitHub, MobGap [41]. The Mobilise-D gait algorithm, therefore, occupies a similar space to SKDH’s gait algorithm as an alternative processing package.

As a matter of fact, at-home gait monitoring has already been implemented in clinical trials, for example, in cancer cachexia phase 1 and 2 studies, as end points designed to support clinical interpretation and decision-making in decentralized trials [42,43]. However, improving accuracy has concrete benefits for clinical translation. In patient populations, small but clinically meaningful changes in gait—such as reductions in stride length in multiple sclerosis [8] or subtle slowing that predicts conversion to Parkinson disease [15]—can be obscured by algorithmic bias or variability, as even small gait deviations can be clinically meaningful. By reducing measurement error, more accurate algorithms increase sensitivity to detect disease progression and treatment response. In clinical trials, this enhanced sensitivity translates into stronger statistical power and potentially smaller required sample sizes. Finally, in the context of real-world monitoring and regulatory acceptance, reliable gait metrics are essential to ensure that longitudinal changes reflect true functional decline or improvement rather than artifacts of the analytic method. This aligns with Food and Drug Administration (FDA) guidance emphasizing the need for trustworthy digital end points in patient-focused drug development [44].

To address the technical challenges mentioned above, reduce bias in the estimation of spatial metrics, and improve overall accuracy, the SKDH-gait algorithm has been overhauled and updated. These updates are significant enough to require new validation beyond what has been previously presented. This work aims to provide a step-by-step overview of the improvements and their validation using data collected from healthy adults and pediatric populations. We first consider each of the constituent blocks of the gait algorithm and examine areas requiring improvement, and then assess these proposed changes. Next, we combine all individual improvements in the full algorithm implementation and validate the updated approach and its effect on the final gait metrics compared with a reference system. Finally, we assess the test-retest reliability of the gait metrics. This paper serves as the first part of the validation work, beginning with healthy participants before transitioning to patient populations in future studies.

## Methods

### Participants and Procedures

Data used in this work for validating the gait algorithm were drawn from the In-Clinic and Natural Gait Observations (ICANGO) [31] and the Monitoring Activity and Gait in Children (MAGIC) [30] studies completed at the Pfizer Innovation Research Lab in Cambridge, Massachusetts.

In MAGIC, a total of 40 healthy ambulatory participants aged 3-17 years were enrolled. Participants were recruited equally from 3 independent age groups as follows: 3-5 years, 6-11 years, and 12-17 years. Informed consent was obtained from the parents or legally acceptable representatives of all participants. Assent was obtained in an age-appropriate manner (verbal assent for children 3-5 years; written assent for children 6-17 years). The study was conducted at the Pfizer Innovation Research Lab in Cambridge, Massachusetts, from 2021 to 2022.

In ICANGO, 2 cohorts of 20 healthy individuals each (40 participants total) over 18 years old (range 25-82 years) were enrolled. Participants did not have any significant health problems, as determined by a medically qualified study doctor during initial intake.

Details of the 2 studies have been previously published [30,31]. Only study procedures relevant to this validation are further elaborated here.

For both studies, participants completed in-laboratory and at-home portions; however, only the in-laboratory portions are considered for this work. Participants in the ICANGO study completed 2 in-laboratory visits spaced approximately 7-14 days apart, while those in the MAGIC study completed only 1 in-laboratory visit. During the in-laboratory visits, participants completed several walking tasks on an instrumented walkway (GAITRite, CIR Systems Inc) while also wearing a set of 6 wearable inertial sensors (OPAL v2, APDM Inc). Only the lumbar-mounted Opal sensor is considered for this work. Pressure data from the GAITRite system were collected at 100 Hz, while acceleration data from the Opal sensors were recorded at 128 Hz. A motion tracking system (SIMI, SIMI Motion Reality Systems) was also used during these in-laboratory walks; however, not all participants had analyzable data from this system. All 3 systems were synchronized via an electronic trigger to ensure precise time alignment.

For the in-laboratory walking tasks, participants completed 3 laps of the 20-foot GAITRite walkway at self-selected natural, slow, and fast walking speeds. Participants in the ICANGO study additionally completed a natural-speed walking task in which a carpet was placed over the GAITRite mat. This walk is considered only in the gait subblock analysis, as it was not included in either study.

### Reference Algorithms

Data provided by the GAITRite mat were analyzed using the ProtoKinetics Movement Analysis Software (PKMAS) to obtain both gait subblock metrics and final gait metrics. Gait subblock metrics were those derived from the intermediate blocks of the gait algorithm, including but not limited to initial contact (IC) and final contact (FC) times. The final gait metrics of interest were limited to cadence, stride time, stride length, and gait speed, as these cover the majority of the temporal and spatial gait metric space.

Center of mass (CoM) displacement can often be used to simplify locomotion trajectories, as in walking, where modeling the CoM as an inverted pendulum aids in estimating spatial gait parameters such as stride length [29]. Reference data for CoM height change were provided by the SIMI system for those participants whose SIMI data were processed; these participants represented only a subset of those in the ICANGO study. Time points (ie, IC and FC events) used to calculate CoM height change were extracted from the PKMAS data.

### The Comparison Gait Algorithm, Gait v2

In this work, the updated SKDH-gait, ie, gait v3, was compared with the last major version of the algorithm implemented in SKDH-gait, named gait v2. As a side note, the original GaitPy implementation is considered gait v1 [7,20], which is not included in the comparison, as it has already been sunset due to obsolescence.

Gait v2 uses continuous wavelet transforms (CWTs) and the inverted pendulum model to analyze the lumbar acceleration signal and generate gait metric estimates. Moving through the algorithm:

- Acceleration was first down-sampled to 50 Hz [20].
- Acceleration frame orientation was corrected to better align with participant’s anatomical vertical, anterior-posterior (AP), and medial-lateral axes using a small-angle accelerometer tilt correction [45].
- Following this orientation correction, a smoothed vertical acceleration signal was obtained by first integrating the vertical acceleration using the cumulative trapezoid rule and then applying the CWT with a Gaussian first-derivative wavelet [21].
- Peaks in this smoothed vertical acceleration with a height above 50% of the SD of the signal were selected as first-pass estimates of the IC events (ie, heel strikes) [21].
- The CWT was then applied to the smoothed vertical acceleration a second time to obtain an estimate of the smoothed jerk [21].
- Peaks in this signal (again with a height >50% of the signal SD) were taken as first-pass estimates of the FC events (ie, toe-offs) [21].

This first step for estimating IC and FC events will be referred to as the “m” method. The scales for the CWT were estimated from step frequency-CWT scale relations derived in previous work [37]. For this relation, an estimate of the step frequency was required, which was obtained using a pass of the algorithm at the scale from the original work [21], defined by a frequency of 1.25 Hz (at 50 Hz, this scale is 8).

Using these first-pass estimates of ICs and FCs, steps are defined by applying the following quality control (QC) rules:

- Loading time (initial double support) should be less than C loading t max stride.
- Stance time should be less than half of t max stride plus the maximum initial double support from the first check.
- Strides should be less than the t max stride.

*t*_max stride_ is the maximum stride time and *C*_loading_ is a loading factor. *t*_max stride_ and *C*_loading_ were set to 2.25 seconds and 0.2, respectively, for the ICANGO study following previous work in gait “v1” [7]. For the MAGIC study, *t*_max stride_ was set to 1.75 seconds and the wavelet scale frequency was set to 1.35 Hz following previous internal work in pediatric populations.

Following the step construction from the ICs and FCs, the CoM height change between consecutive ICs was estimated by double-integrating the vertical acceleration. This CoM height change was then used in the inverted pendulum model to estimate step length [22], as described below.



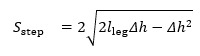



where *S*_step_ is the step length, *l*_leg_ is the leg length estimated from 0.53 × height [35], and Δ*h* is the CoM change in height. Step time was defined as the interval between consecutive ICs, and stride time as the sum of 2 consecutive step times. Stride length was defined as the sum of consecutive step lengths. Gait speed was calculated as stride length divided by stride time, and cadence was defined as the number of steps taken per minute.

Cadence = (60/*t*_Step_)

where *t*_Step_ is the step time.

### Comparison Contact Event Algorithms

In addition to the full comparison algorithm (gait v2), we implemented several previously published methods for estimating IC and FC events to provide additional benchmarks for our proposed method. These comparator methods are summarized in Table 1.

**Table 1:** Additional contact event methods implemented to compare the original method used in gait v2 (“m” method) and the method proposed in this work.

Method	Events	Description	Reference
m (gait v2)	IC^a^/FC^b^	Peaks in CWT^c^-smoothed vertical acceleration and jerk	[21]
B	IC	Peaks in filtered AP^d^ acceleration data	[46]
G	IC/FC	Zero-crossings in FIR^e^-filtered AP acceleration data with heuristic rules	[47]
z-cross	IC	Zero-crossings in the filtered AP acceleration data	[22]
z-peak	IC	Peaks preceding zero-crossings in the filtered AP acceleration data	[22]
Pe	IC/FC	Stationary wavelet transform and identification using AP and vertical acceleration	[48,49]
Sk	IC	CWT of acceleration magnitude; peaks in CWT coefficients accounting for varying step frequency	[50]

^a^IC: initial contact.

^b^FC: final contact.

^c^CWT: continuous wavelet transform.

^d^AP: anterior-posterior (ie, forward-backward) axis.

^e^FIR: finite impulse response.

### Proposed Gait Algorithm

#### Overview

**Figure 1:**
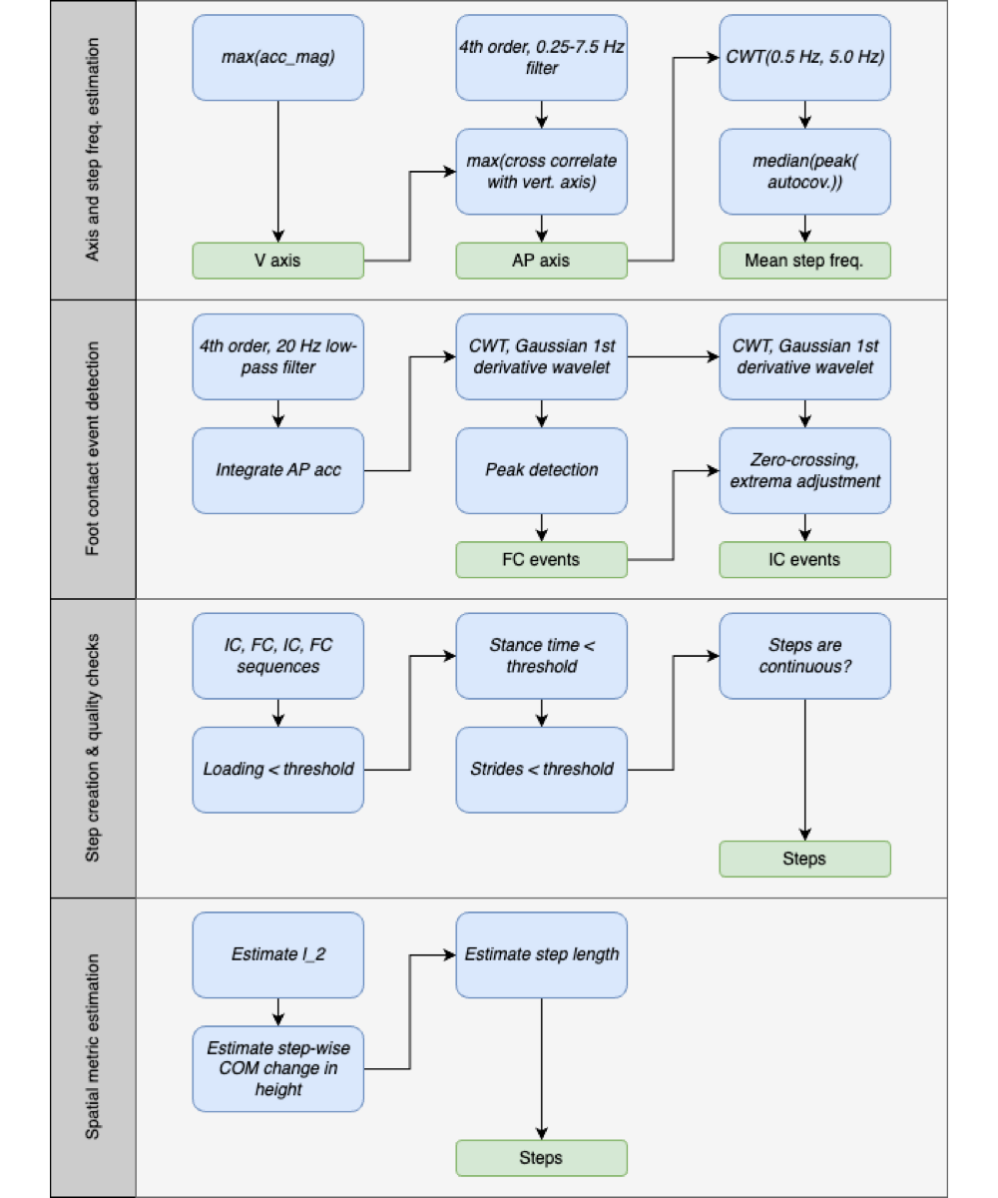
Simplified diagram showing the full updated gait algorithm. AP: anterior-posterior; CoM: center of mass; CWT: continuous wavelet transform; FC: final contact; IC: initial contact.

Building on gait v2, we improved key components for estimating gait metrics from lumbar acceleration, resulting in the updated gait v3 algorithm. Although all data from the ICANGO and MAGIC studies were used in the final analysis of gait metrics, the subblock improvements were developed using a random ≈50% train-test split within each study. This yielded a training set of n=33 (14/19 from ICANGO/MAGIC) and a test set of n=47 (26/21 from ICANGO/MAGIC). Note that, in gait v3, acceleration data are not downsampled. Figure 1 presents the updated gait algorithm.

#### Accelerometer Axis and Mean Step Frequency Estimation

The first major subblock in the proposed gait algorithm estimates (1) the accelerometer axis most closely aligned with the participant’s AP axis and (2) the mean step frequency for the entire walking bout. Identifying the AP axis is essential for applying the small-angle orientation correction and for subsequent contact event detection, which relies on the AP signal. Estimating the mean step frequency is also important, as it informs the dynamic QC thresholds based on walking speed and determines the appropriate CWT scale for smoothing the acceleration signal.

To estimate the AP axis, we first identified the vertical axis as the one with the largest absolute mean acceleration value. The acceleration signal was then filtered using a fourth-order band-pass Butterworth filter (0.25-7.5 Hz) to isolate walking-related components. After filtering, the AP axis was defined as the axis showing the highest cross-correlation with the vertical axis.

To estimate the mean step frequency, we computed CWTs of the AP-axis acceleration across 10 scales spanning 0.5-5.0 Hz using a Gaussian first-derivative wavelet. These 10 CWT coefficients were summed to produce a single signal representing the “CWT power” in that frequency range. A windowed (5 seconds, 50% overlap) autocovariance was then calculated, and for each window, the first peak was extracted. The median of these first peaks was defined as the mean step time, which was inverted to obtain the mean step frequency.

#### Foot Contact Event Detection

**Figure 2:**
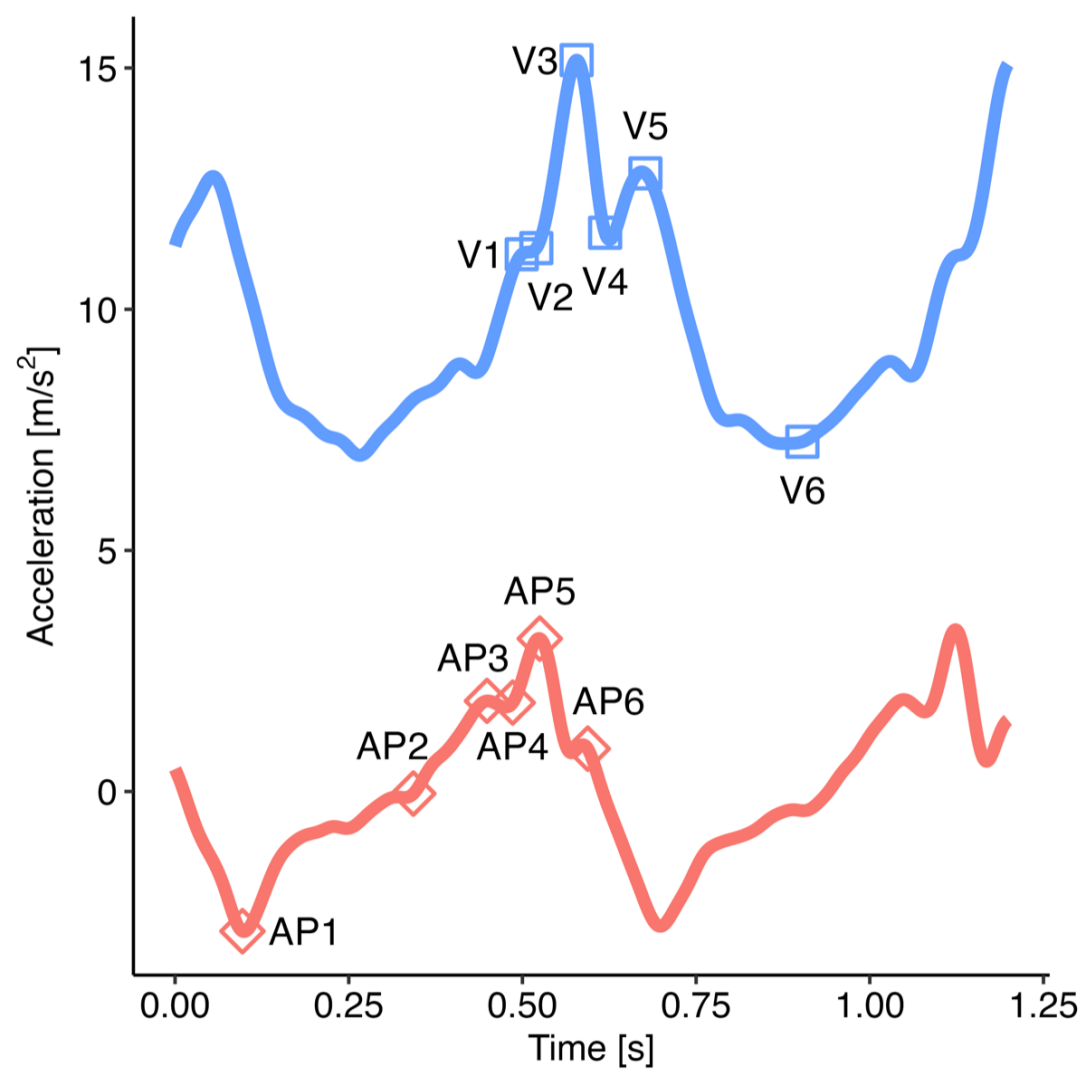
Synthetic example acceleration traces for the vertical (V) and anterior-posterior (AP) axes. Several key points in both traces are labeled following previous convention [46].

While the “m” method (The Comparison Gait Algorithm, Gait v2 [21]) for estimating contact events is generally reliable and yields good agreement for temporal gait metrics (ie, stride time), it is not the most accurate for determining the exact timing of these events [51]. Figure 2 shows the vertical and AP acceleration from a lumbar device, with several key points labeled.

The issue arises from the smoothing of the vertical acceleration, which is very effective at locating point “V3” of the vertical acceleration [21]. However, this point does not correspond to the actual moment of IC, which is instead point “AP5” [22,48]. Therefore, we propose a new method that utilizes the AP-axis acceleration and dynamically adjusts the wavelet scale based on the mean step frequency for the walking bout.

First, we calculated the CWT scales based on the estimated mean step frequency per



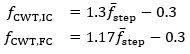



where *f*_CWT_ is the Gaussian first derivative wavelet central frequency for the IC and FC detection, and 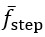 is the estimated mean step frequency. The relations were obtained by performing a scale sweep on the training data and optimizing both the *F*_1_-score and the root-mean-square error using a cost function [37]. We note that the generalizability of these relations to pathological cohorts should be further explored in future work. Although the step frequency-wavelet frequency relation only informs the CWT-based filtering based on walking speed, and therefore should not be affected by pathologies, this assumption should be confirmed.

To remove any very high-frequency noise, acceleration was filtered with a 20 Hz cutoff using a fourth-order Butterworth filter. Next, FC events were obtained by first integrating the filtered AP acceleration to compute the AP velocity, and then applying the CWT (Gaussian first-derivative wavelet) using the scale corresponding to the central frequency from the above equations. Peaks in the resulting CWT coefficients correspond to FC events. To minimize false positives, we applied 2 constraints to the peak detection: first, the prominence had to be greater than 0.6σ*_Wn_*, and second, the peak distance had to be greater than 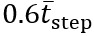, where σ*_Wn_* is the SD of the CWT coefficients and 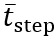 is the estimated mean step time. These constraints were added after exploration of the training dataset during method development, based on visual inspection and experience, to ensure physiologically reasonable detections.

To obtain IC estimates, we first applied the CWT to the filtered AP acceleration using the scale corresponding to the estimated frequency, producing a smoothed AP jerk signal. Local minima and maxima were then identified using a prominence constraint of 0.6σ*_Wn_* and a distance constraint 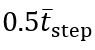. Finally, for each FC event, we searched backward to locate the corresponding IC event, defined as the point halfway between the preceding zero-crossing (positive to negative) and the preceding local minimum. This approach—rather than relying solely on local extrema—helps account for signal perturbations and flatter peaks that can occur during slow walking.

#### Step Creation and Physiological Thresholds

With the IC and FC event estimates obtained, we proceeded to stride creation and QC using physiological thresholds. The same basic rules as in gait v2, described above in “The Comparison Gait Algorithm, Gait v2” section, were applied.

**Figure 3:**
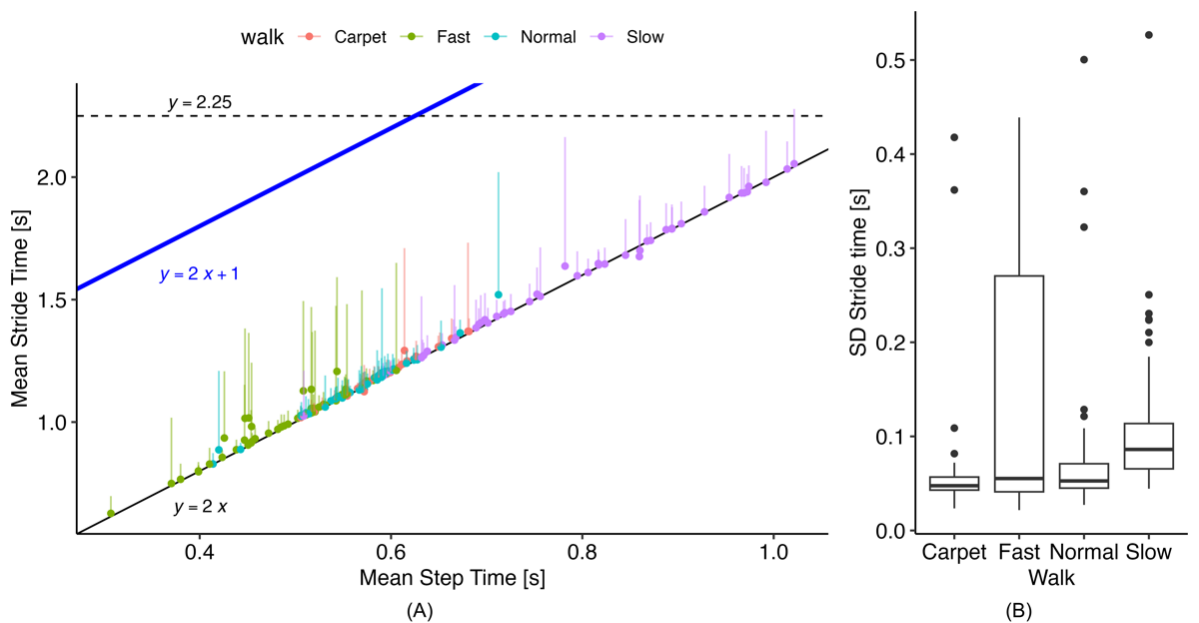
GAITRite/PKMAS reference data from the training data set (N=33), where each ICANGO participant completed each of the 4 walks, and MAGIC participants completed the fast, normal, and slow walks. (A) Relationship between mean step time and mean stride time. The original quality control threshold is the dashed line, y=2.25, the new dynamic threshold is y=2x+1. (right) Stride time SD per walking task. ICANGO: In-Clinic and Natural Gait Observations; MAGIC: Monitoring Activity and Gait in Children; PKMAS: ProtoKinetics Movement Analysis Software.

These rules require defining 2 parameters: *t*_max stride_ and *C*_loading_. While gait v2 uses static thresholds for these values, we explored dynamic functions based on the estimated mean step time. Figure 3 shows the relationship between mean step time and stride time from the reference data, which generally follows the expected 1:2 relation. From this plot, we observed that the original static threshold of 2.25 seconds did not adequately cover the full range of walking speeds, particularly during fast walking, where several strides could occur before reaching 2.25 seconds. Examining the SD of stride times, we found a maximum of approximately 0.5 25 seconds. Therefore, we set a new dynamic threshold based on the following:



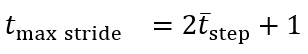



This provides a threshold set at least 2 SDs above the expected mean stride time.

**Figure 4:**
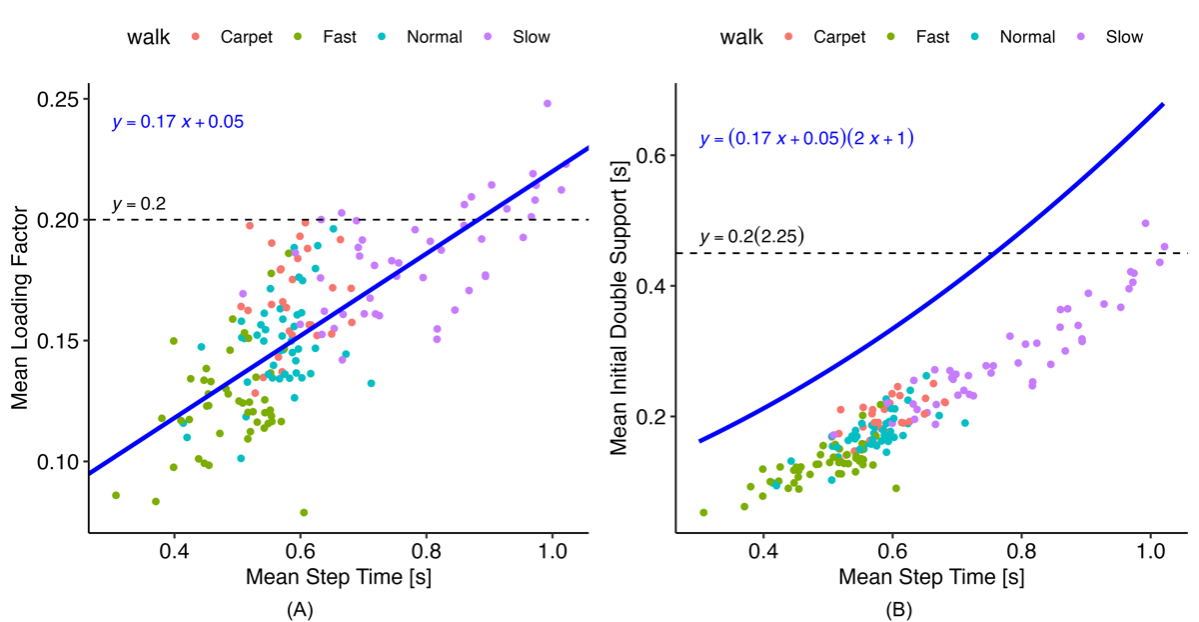
GAITRite/PKMAS reference data from the training data set (N=33), where each ICANGO participant completed each of the 4 walks, and MAGIC participants completed the fast, normal, and slow walks. (A) Relationship between mean step time and mean loading factor across various walking speeds. Original factor of 0.2 is the dashed line. The new dynamic relation for loading factor, y=0.17x+0.05, is the blue line. (B) Relationship between mean step time and mean initial double support time. The dashed line is the original threshold of 0.2(2.25)=0.45. The blue line is the new threshold based on the maximum stride time and loading factor dynamic relationships. ICANGO: In-Clinic and Natural Gait Observations; MAGIC: Monitoring Activity and Gait in Children; PKMAS: ProtoKinetics Movement Analysis Software.

Figure 4 illustrates the relationship between mean step time and the loading factor derived from the reference PKMAS gait data. The data show that the loading factor is not constant across walking speeds, decreasing noticeably as walking speed increases. To account for this, we implemented the following relation to calculate the loading factor:



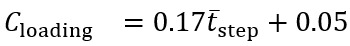



The coefficients for this relation were obtained using simple linear regression on the reference data. This approach provided a much-improved limit for initial double support times compared with the original threshold (Figure 3), producing a more consistent gap between the mean initial double support time and the threshold across walking speeds. The blue line in Figure 3 represents the upper limit for allowable initial double support values and is therefore positioned above the mean to accommodate the variability of initial double support times within a walking bout.

Using these new threshold calculations, steps were created by identifying groups consisting of 1 IC and 2 FCs (opposite and same side) that satisfied the dynamic QC thresholds. We also ensured continuity, such that the same-side FC of one step served as the opposite-side FC for the subsequent step. Once steps were allocated in this manner, temporal gait metrics were computed for each step or for continuous steps, as appropriate.

#### Inverted Pendulum Model and Spatial Gait Metrics

**Figure 5:**
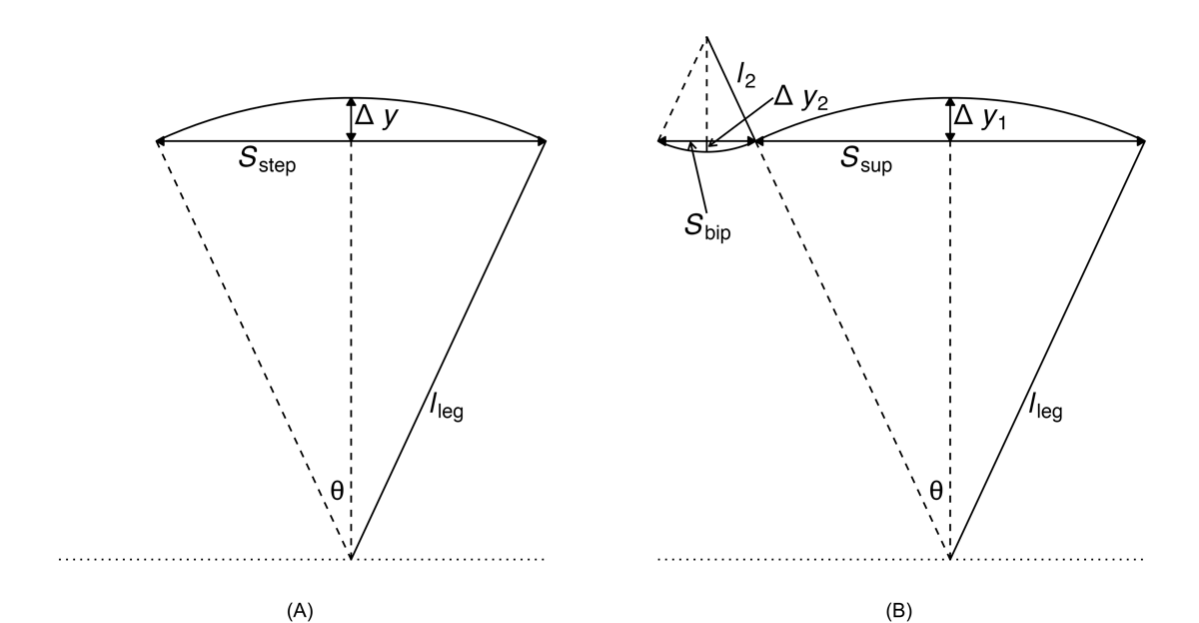
Inverted pendulum models [29]. (A) Inverted pendulum model 1, where there is only an inverted pendulum. The only unknown is S_step, which allows for easy solving. Used in gait v2. (B) Inverted pendulum model 2, where there is both a normal and inverted pendulum. S_bip is the step length during initial double support, S_sup is the step length during single support. This model has 3 unknowns: S_bip, l_2, and S_sup.

Two inverted pendulum models are depicted in Figure 5. Model 1 consists of a single inverted pendulum, whereas model 2 incorporates both a normal and an inverted pendulum. To improve spatial metric estimation in the proposed gait algorithm, we utilized the inverted pendulum model 2 [29]. This model can be described by the following equations [22,29]:



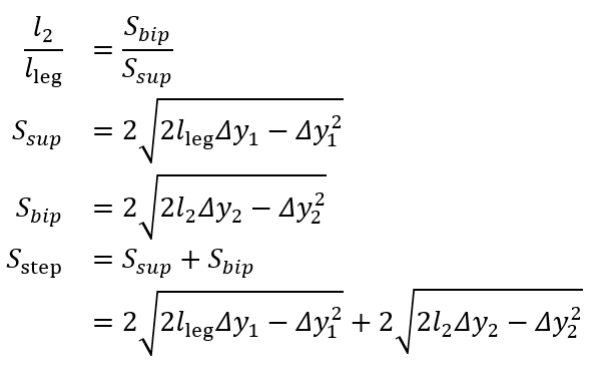



One unknown remained on the right-hand side, *l*_2_. To address this, we used the available temporal metrics to establish a relationship between the length ratio and the time ratio of these 2 gait phases, as follows:



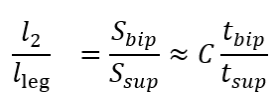



where *t_bip_* and *t_sup_* represent the initial double support and single support durations for a step, respectively.

**Figure 6:**
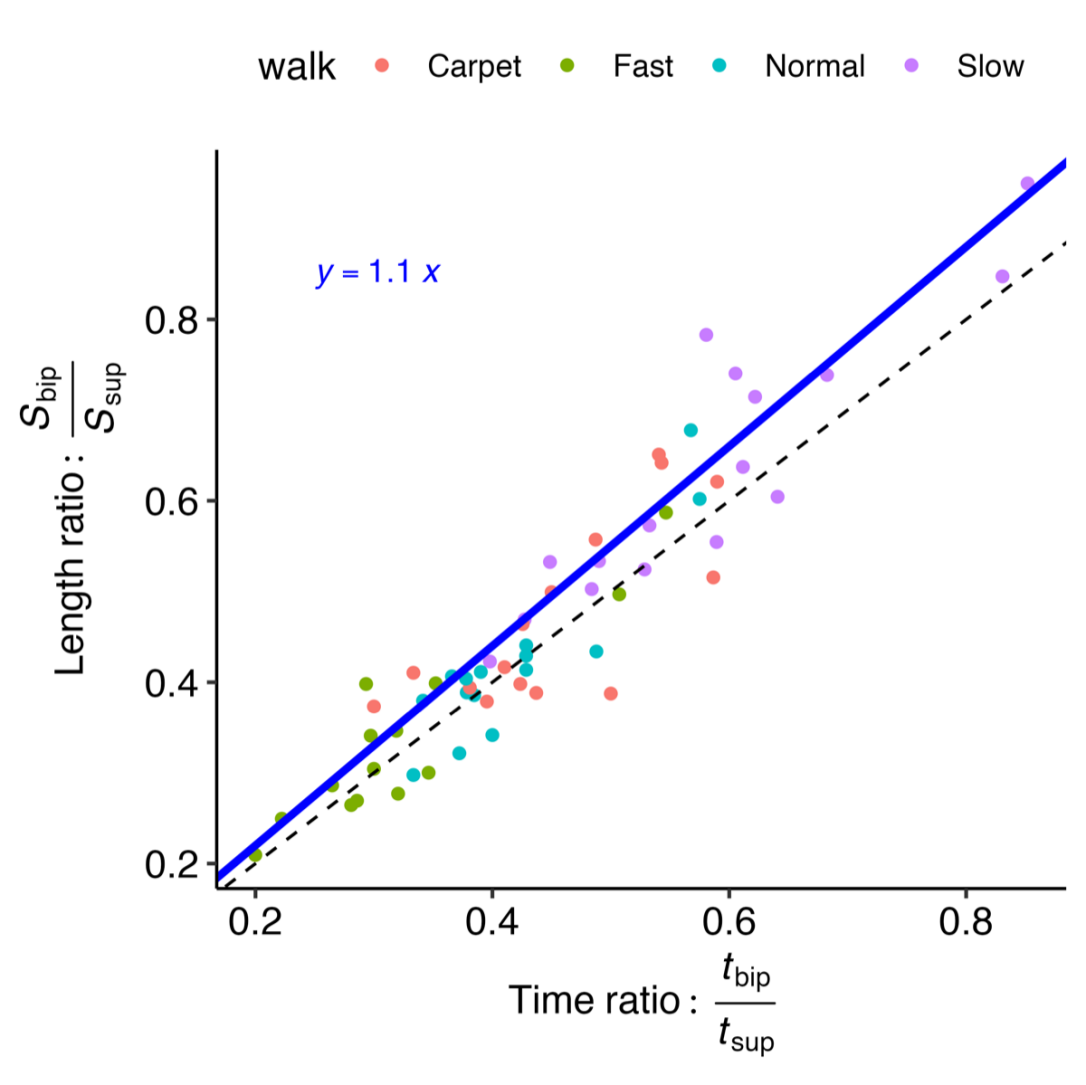
Relationship between the time and length ratios.

Figure 6 illustrates the experimental relationship described by the above equations, derived from reference PKMAS and SIMI data. From this training data, we obtained *C*=1.1 such that 
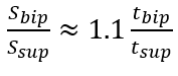
. Using this relationship, we estimated *l*_2_ as follows:

*l*_2_ = 1.1*l*_leg_(*t_bip_/t_sup_*)

which enabled the calculation of the step length estimate using model 2. To minimize the variability in per-step estimations of the length-to-time ratio, we computed *l*_2_ using the median *t_bip_* and *t_sup_* values across individual bouts of gait.

### Data and Statistical Analysis

#### Overview of the Analysis

The analyses were conducted in 2 parts: first, we computed values for each gait subblock described above and compared them with ground truth as well as the comparison algorithms; second, we computed final gait metrics for the proposed method (gait v3) and the comparison method (gait v2) and compared these with the reference.

For all analyses, when summarizing values across a walking task, the median value across all steps was used. When summarizing values across visits (for ICANGO), the average of the 2 visits was used. Intraclass correlation coefficients (ICCs) were used to evaluate agreement, with ICC≤0.4 indicating “poor,” <0.4-0.59 indicating “moderate,” 0.6-0.74 indicating “good,” and 0.75-1.0 indicating “excellent” agreement [52].

#### Gait Subblock Performance Analysis

For the first part of the analysis, ICANGO and MAGIC participants were randomly divided into training and testing sets within each study (without accounting for age, gender, or other covariates) to develop the proposed method. This resulted in n=33 in the training set (14/19 from ICANGO/MAGIC), and n=47 in the test set (26/21 from ICANGO/MAGIC). Results for the gait subblocks are presented using the test set data. Additional results are provided in Multimedia Appendix 1. Where appropriate, these results used outputs from the ground truth methods to isolate the effects of individual subblocks—for example, CoM height change estimation used reference method contact events as bounds for integrating acceleration.

For the comparison of mean step time estimation, we used ICC (2-way random effects, absolute agreement), Pearson correlation coefficients (Pearson ), and repeated measures correlation coefficients (RMCCs) to account for within-participant correlation (ie, multiple walking speed tasks per participant). Analysis of covariance was used to statistically adjust for interindividual variability [53,54].

To assess the performance of the gait subblock values, we considered both per-step values and median values across walking tasks. For IC and FC detection, performance was evaluated using sensitivity, precision, *F*_1_-score, and mean absolute error (MAE) relative to the reference system. To determine true and false positives and negatives, each contact event in the reference system was matched to a corresponding event within a 0.3-second window (ie, 0.15 seconds on each side). Previous studies have used smaller windows (eg, 50 ms [37]), but such narrow windows would have excluded many events and artificially increased MAE. Events occurring outside the GAITRite mat area were not counted as false positives. MAE was computed only for contact events corresponding to a reference contact event. Performance metrics were assessed both visually and using linear mixed-effects regression (LMER) models to account for interactions between method, walking task, and age group, while controlling for within-participant correlation using a participant-specific random effect.

#### Gait Metric Performance Analysis

For the second part of the analysis, the full set of ICANGO and MAGIC participants was used, and gait metrics were compared between the 2 algorithm versions and the reference results. Gait v2 and gait v3 were computed using SKDH version 0.13.0. The SKDH code is available on GitHub [55]. The analysis focused on 4 common gait metrics—cadence, stride time, stride length, and gait speed—which together cover both temporal and spatial gait parameters.

To comprehensively evaluate the accuracy of gait metrics derived from gait v3 against both the reference system (PKMAS) and gait v2, we applied a hierarchical statistical framework to distinguish primary and secondary outcomes. Primary analyses were defined as agreement with reference values across walking speeds and were assessed using (1) Pearson correlation coefficients (*r*) to evaluate whether the algorithm preserved the relative ranking of individuals, and (2) ICC (2-way random effects, absolute agreement) to evaluate whether algorithm outputs were in absolute agreement with the reference. These measures directly capture the clinical interpretability of gait v3 relative to gold-standard assessments. Secondary analyses focused on evaluating whether gait v3 reduced systematic bias relative to gait v2. To this end, LMER models were used to compare PKMAS, gait v2, and gait v3 across walking speeds and age groups (>18, 12-17, and <12 years) within a comprehensive modeling framework. The 4 gait metrics (cadence, stride time, stride length, and gait speed) were modeled as outcomes, with algorithm, walk type, age group, and the interactions algorithm × walk type and algorithm × age group included as fixed effects. Participant-specific random intercepts were included to account for repeated measures. Pairwise contrasts of estimated means were calculated, with *P* values adjusted for multiple comparisons using the false discovery rate.

In the ICANGO study, where participants had 2 in-laboratory visits, we additionally assessed test-retest reliability using ICC (2-way random effects, absolute agreement), Pearson correlation coefficients, and RMCCs.

The following R packages (R Foundation) were used: rmcorr (version 0.6.0), lme4 (version 1.1-35.3), lmerTest (version 3.1.3), emmeans (version 1.10.1), rstatix (version 0.7.2), psych (version 2.4.3), and BlandAltmanLeh (version 0.3.1). R version 4.3.3 was used.

### Ethics Considerations

Ethics approval for the MAGIC protocol was obtained from the Advarra Institutional Review Board (protocol number: Pro00047861). All participants provided informed consent. The ICANGO study was reviewed and approved by the Advarra Institutional Review Board (protocol number: Pro00043100).

## Results

### Mean Step Time Estimation

The agreement between the gait v3 mean step-time estimates and the reference method is presented in Table 2. The overall agreement was excellent, with all ICC values exceeding 0.91 and Pearson *r* values above 0.92. Repeated-measures analysis yielded similar findings, demonstrating a high RMCC of 0.98.

**Table 2:** Agreement for mean step frequency estimation between gait v3 and PKMAS^a^ across different walking tasks^b^.

Walk	PKMAS (Hz), mean (SD)	Gait v3 (Hz), mean (SD)	*r*^c^ (95% CI)	ICC^d^ (95% CI)	RMCC^e^ (95% CI)
Normal	1.86 (0.23)	1.84 (0.23)	0.98 (0.97-0.99)	0.98 (0.95-0.99)	0.98 (0.97-0.99)
Fast	2.08 (0.28)	2.12 (0.29)	0.92 (0.86-0.96)	0.91 (0.83-0.95)	0.98 (0.97-0.99)
Slow	1.40 (0.26)	1.39 (0.25)	0.99 (0.97-0.99)	0.98 (0.97-0.99)	0.98 (0.97-0.99)
Carpet	1.69 (0.13)	1.69 (0.14)	0.94 (0.87-0.97)	0.94 (0.87-0.97)	0.98 (0.97-0.99)

^a^PKMAS: ProtoKinetics Movement Analysis Software.

^b^This analysis is based on the test set.

^c^Pearson correlation coefficient.

^d^ICC: intraclass correlation coefficient.

^e^RMCC: repeated measures correlation coefficient.

### Initial and Final Contact Estimation

**Figure 7:**
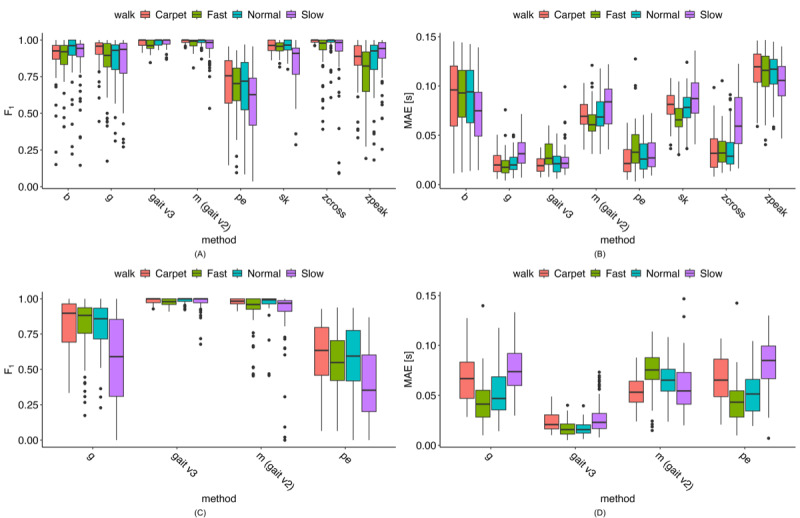
Test-set accuracy results for various initial and final contact estimation methods from data across visits and walking tasks (N=47). See Table 1 for the list of algorithms and references. (A) Initial contact F1-score results. (B) Initial contact mean absolute error (MAE) results. (C) Final contact F1-score results. (D) Final contact MAE.

The accuracy of the proposed and comparison methods for IC/FC estimation relative to the reference methods—along with several additional literature-based methods (see Table 1)—is shown in Figure 7A. The gait v3 method achieved among the highest *F*_1_-scores, with median values above 0.9; the only comparable approach was the “m” method (gait v2). When comparing MAE across methods (Figure 7B), gait v3 again performed among the best for IC estimation, with only the “g” method showing similar MAE values. The “pe” method approached this performance but generally produced higher errors. Notably, gait v3 showed an approximately 50% reduction in MAE compared with the “m” (gait v2) method. Full LMER model results comparing each algorithm with the gait v3 method are provided in Multimedia Appendix 1.

When comparing FC method *F*_1_-scores (Figure 7C), none of the literature-based methods approached the performance of either the “m” (gait v2) or gait v3 methods. Although both “m” and gait v3 exhibited some low *F*_1_-score outliers, gait v3 had noticeably fewer outliers, and those that did occur were generally higher than the outliers observed for the “m” method. For MAE (Figure 7D), the gait v3 method had the lowest values, with median MAE typically about 50% lower than any of the other methods.

Exploring the outliers reveals that most are not consistent across methods for either IC or FC estimation; rather, the outliers for different methods arise from different participant-walk tasks. Notably, for the gait v3 method, all *F*_1_-score outliers remain above 0.81 for IC events and above 0.65 for FC events.

### Gait Metrics

Using data from the ICANGO and MAGIC studies, we evaluated the performance of the gait v2 and v3 algorithms against reference PKMAS values. Table 3 summarizes the algorithm and reference metrics, along with agreement statistics (Pearson correlation coefficients and ICCs) for each gait variable and walking task. Correlation coefficients were generally high and comparable between gait v2 and gait v3, with the lowest Pearson correlation of 0.73 for stride time during the slow walk using gait v2, and 0.82 for stride length during the slow walk using gait v3. By contrast, ICC values showed a consistent improvement with gait v3: while gait v2 exhibited a minimum ICC of 0.50 (gait speed, fast walk), gait v3’s lowest ICC was 0.81 (stride length, slow walk) across all metrics and walking tasks. The most notable improvements in ICC values were observed for the spatial metrics—stride length and gait speed. Highlighting a few specific instances, stride time during the slow walk showed a substantial increase, with Pearson *r* improving from 0.73 in v2 to 1.00 in v3, and ICC increasing from 0.70 to 1.00. Similarly, gait speed showed marked gains: during the fast walk, ICC improved from 0.50 to 0.84, and during the slow walk, from 0.71 to 0.90. These examples illustrate the consistent enhancement in agreement achieved with the gait v3 algorithm.

**Table 3:** Comparison results for gait metrics across walking speed (N=80).

Metric and walk		PKMAS^a^, mean (SD)	Gait v2, mean (SD)	Gait v3, mean (SD)	Gait v2, *r*^b^ (95% CI)	Gait v3, *r* (95% CI)	Gait v2, *r* (95% CI)	Gait v3, ICC^c^
**Cadence (step/minute)**								
	Normal	111.45 (13.90)	109.70 (13.50)	110.22 (13.97)	0.99 (0.98-0.99)	0.99 (0.99-1.00)	0.98 (0.91-0.99)	0.99 (0.96-1.00)
	Fast	129.25 (18.41)	123.76 (15.18)	127.83 (18.39)	0.85 (0.78-0.90)	0.99 (0.98-0.99)	0.80 (0.59-0.89)	0.98 (0.97-0.99)
	Slow	83.15 (15.72)	84.65 (13.90)	82.26 (15.48)	0.93 (0.89-0.96)	1.00 (0.99-1.00)	0.93 (0.89-0.96)	1.00 (0.99-1.00)
**Stride time (seconds)**								
	Normal	1.10 (0.13)	1.11 (0.12)	1.10 (0.13)	0.99 (0.99-0.99)	1.00 (1.00-1.00)	0.99 (0.96-0.99)	1.00 (0.99-1.00)
	Fast	0.95 (0.12)	0.98 (0.11)	0.96 (0.14)	0.89 (0.83-0.93)	0.89 (0.84-0.93)	0.86 (0.73-0.92)	0.88 (0.83-0.92)
	Slow	1.50 (0.31)	1.41 (0.22)	1.52 (0.31)	0.73 (0.61-0.82)	1.00 (1.00-1.00)	0.75 (0.60-0.85)	1.00 (0.99-1.00)
**Stride length (m)**								
	Normal	1.24 (0.21)	1.11 (0.21)	1.22 (0.21)	0.89 (0.83-0.93)	0.85 (0.77-0.90)	0.74 (-0.01-0.91)	0.84 (0.77-0.90)
	Fast	1.43 (0.25)	1.26 (0.24)	1.41 (0.25)	0.89 (0.83-0.93)	0.88 (0.82-0.92)	0.71 (-0.05-0.90)	0.88 (0.82-0.92)
	Slow	1.00 (0.22)	0.87 (0.17)	1.03 (0.19)	0.85 (0.77-0.90)	0.82 (0.73-0.88)	0.68 (0.03-0.87)	0.81 (0.71-0.87)
**Gait speed (m/s)**								
	Normal	1.14 (0.19)	1.00 (0.19)	1.12 (0.20)	0.87 (0.81-0.92)	0.83 (0.74-0.88)	0.69 (-0.05-0.89)	0.82 (0.73-0.88)
	Fast	1.51 (0.22)	1.28 (0.20)	1.48 (0.22)	0.79 (0.69-0.86)	0.84 (0.77-0.90)	0.50 (-0.10-0.80)	0.84 (0.76-0.89)
	Slow	0.69 (0.21)	0.63 (0.16)	0.70 (0.18)	0.78 (0.67-0.85)	0.90 (0.85-0.94)	0.71 (0.49-0.83)	0.90 (0.85-0.93)

^a^PKMAS: ProtoKinetics Movement Analysis Software.

^b^Pearson correlation coefficient.

^c^ICC: intraclass correlation coefficient.

**Figure 8:**
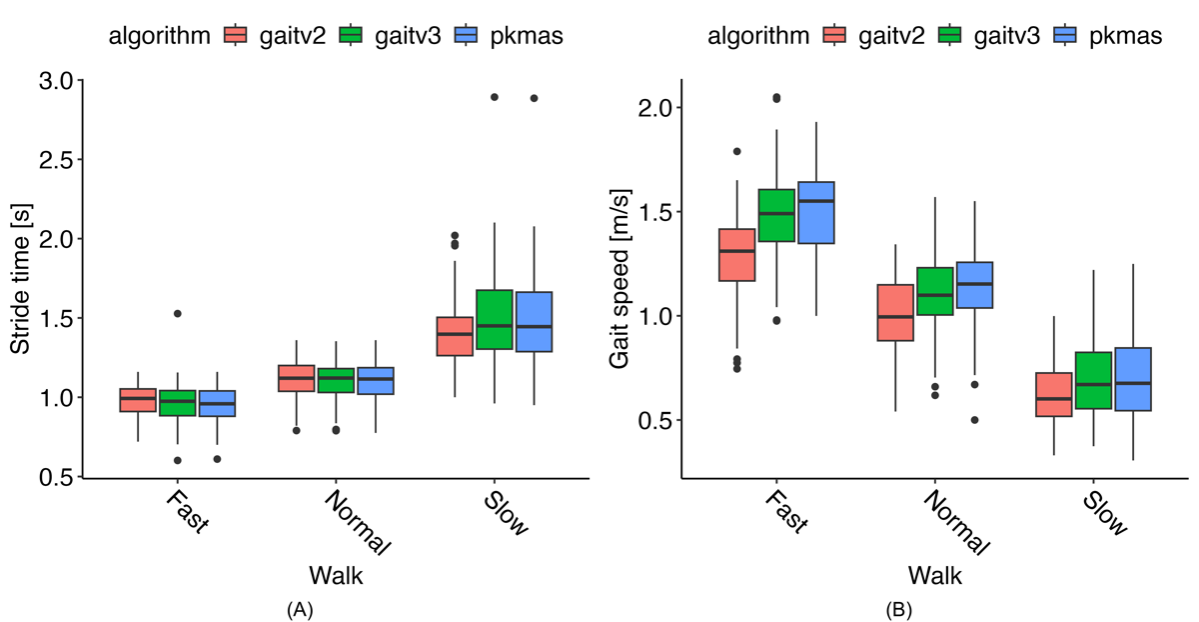
Gait metric results across walking tasks from reference, comparison, and proposed algorithms (N=80). Median values are taken across steps per walking task and mean values across visits. (A) Stride time results. (B) Gait speed results. PKMAS: ProtoKinetics Movement Analysis Software.

This improved agreement is illustrated in Figure 7, where stride time results are generally consistent across methods and walking speeds. However, Figure 8B shows that gait v2 aligns less closely with PKMAS results across all 3 gait speeds. Bland-Altman plots indicate that this difference reflects a mostly consistent bias, with gait v2 generally underestimating gait speed (see Multimedia Appendix 1).

Table 4 presents the pairwise contrasts of estimated means from the LMER models across walking tasks. When comparing gait v2 with PKMAS, most metrics showed significant differences (*P*<.007), except for cadence during the normal and slow walks, and stride time during the normal and fast walks. By contrast, gait v3 showed no significant differences from PKMAS for any metric across all walking tasks (the smallest *P* value was .14). Direct comparison of v2 and v3 revealed that the metrics where v2 differed from PKMAS also exhibited significant differences between v2 and v3 (*P*<.009). These significant differences in gait speed indicate that v2 was 0.064-0.199 m/s slower than v3, and 0.065-0.230 m/s slower than PKMAS.

**Table 4:** Mixed-effects model contrast estimates (and 95% CIs) across walk types for gait metrics (N=80)^a^.

Metric and walk			PKMAS^b^ – gait v2					PKMAS – gait v3					Gait v2 – gait v3		
			Difference (95% CI)		*P* value		Difference (95% CI)			*P* value		Difference (95% CI)			*P* value
**Cadence (step/minute)**															
	Normal	1.839 (–1.80 to 5.48)		.65		1.182 (–2.46 to 4.82)			.65		–0.657 (–4.30 to 2.98)			.66	
	Fast	5.568 (1.93 to 9.21)		*<.001*		1.361 (–2.28 to 5.00)			.37		–4.207 (–7.85 to 0.57)			*.009*	
	Slow	–1.368 (–5.03 to 2.29)		.55		0.726 (–2.93 to 4.38)			.63		2.094 (–1.56 to 5.74)			.51	
**Stride time (seconds)**															
	Normal	–0.006 (–0.06 to 0.05)		.98		–0.007 (–0.06 to 0.05)			.98		–0.001 (–0.06 to 0.06)			.98	
	Fast	–0.024 (–0.08 to 0.03)		.62		–0.012 (–0.07 to 0.04)			.62		0.011 (–0.04 to 0.07)			.62	
	Slow	0.092 (0.04 to 0.15)		*<.001*		–0.015 (–0.07 to 0.04)			.52		–0.107 (–0.16 to –0.05)			*<.001*	
**Stride length (m)**															
	Normal	0.136 (0.10 to 0.18)		*<.001*		0.021 (–0.02 to 0.06)			.21		–0.115 (–0.16 to –0.07)			*<.001*	
	Fast	0.174 (0.13 to 0.22)		*<.001*		0.018 (–0.02 to 0.06)			.29		–0.156 (–0.20 to –0.12)			*<.001*	
	Slow	0.133 (0.09 to 0.17)		*<.001*		–0.025 (–0.07 to 0.02)			.14		–0.158 (–0.20 to –0.12)			*<.001*	
**Gait speed (m/s)**															
	Normal	0.135 (0.08 to 0.19)		*<.001*		0.024 (–0.03 to 0.08)			.28		–0.111 (–0.16 to –0.06)			*<.001*	
	Fast	0.230 (0.18 to 0.28)		*<.001*		0.030 (–0.02 to 0.08)			.18		–0.199 (–0.25 to –0.15)			*<.001*	
	Slow	0.065 (0.01 to 0.12)		*.007*		0.000 (–0.05 to 0.05)			.99		–0.064 (–0.12 to –0.01)			*.007*	

^a^*P* values <.05 are in italics. *P* values are corrected for multiple comparisons within each gait metric.

^b^PKMAS: ProtoKinetics Movement Analysis Software.

Table 5 shows a similar pattern, with significant differences between v2 and PKMAS for all age groups in stride length and gait speed. While v3 also showed some differences from PKMAS in these metrics, the discrepancies were smaller than those observed for v2. Direct comparison between v2 and v3 revealed that v2 had shorter stride lengths and slower gait speeds, with differences ranging from 0.134 to 0.156 m for stride length and 0.098 to 0.162 m/s for gait speed.

**Table 5:** Mixed-effects model contrast estimates (and 95% CIs) across age groups for gait metrics (N=80).^a^

Metric and age group			PKMAS^b^ – gait v2					PKMAS – gait v3					Gait v2 – gait v3		
			Difference (95% CI)		*P* value		Difference (95% CI)			*P* value		Difference (95% CI)			*P* value
**Cadence (step/minute)**															
	>18	1.571 (–1.34 to 4.48)		.48		1.202 (–1.70 to 4.10)			.48		–0.368 (–3.27 to 2.53)			.76	
	<12	2.430 (–1.23 to 6.09)		.33		1.224 (–2.43 to 4.88)			.43		–1.206 (–4.86 to 2.45)			.43	
	12-17	2.039 (–2.68 to 6.76)		.67		0.843 (–3.88 to 5.57)			.67		–1.196 (–5.92 to 3.53)			.67	
**Stride time (seconds)**															
	>18	0.001 (–0.04 to 0.05)		.94		–0.010 (–0.05 to 0.03)			.90		–0.011 (–0.06 to 0.03)			.90	
	<12	0.034 (–0.02 to 0.09)		.21		–0.010 (–0.07 to 0.05)			.66		–0.045 (–0.10 to 0.01)			.17	
	12-17	0.027 (–0.05 to 0.10)		.57		–0.014 (–0.09 to 0.06)			.63		–0.041 (–0.11 to 0.03)			.54	
**Stride length (m)**															
	>18	0.161 (0.13 to 0.19)		*<.001*		0.023 (–0.01 to 0.06)			.09		–0.139 (–0.17 to –0.11)			*<.001*	
	<12	0.094 (0.05 to 0.14)		*<.001*		–0.062 (–0.10 to –0.02)			*<.001*		–0.156 (–0.20 to –0.12)			*<.001*	
	12-17	0.188 (0.13 to 0.24)		*<.001*		0.054 (0.00 to 0.11)			*.01*		–0.134 (–0.19 to –0.08)			*<.001*	
**Gait speed (m/s)**															
	>18	0.149 (0.11 to 0.19)		*<.001*		0.035 (–0.01 to 0.08)			.05		–0.115 (–0.16 to –0.07)			*<.001*	
	<12	0.115 (0.06 to 0.17)		*<.001*		–0.047 (–0.10 to 0.01)			*.04*		–0.162 (–0.22 to –0.11)			*<.001*	
	12-17	0.165 (0.09 to 0.24)		*<.001*		0.067 (–0.00 to 0.14)			*.02*		–0.098 (–0.17 to –0.03)			*<.001*	

^a^*P* values <.05 are in italics. *P* values are corrected for multiple comparisons within each gait metric.

^b^PKMAS: ProtoKinetics Movement Analysis Software.

### Test-Retest Reliability

We assessed the test-retest reliability of the reference, comparison, and proposed methods across walking speeds and gait metrics. Overall, the results in Table 6 show excellent ICC agreement, with similar values across methods. Nearly all ICCs exceeded 0.75, except for gait v2 slow-walk cadence and gait v3 fast-walk stride time. The lower agreement for gait v3 stride time during the fast walk was influenced by an outlier, likely caused by accelerometer misalignment affecting the AP acceleration during the task. By contrast, gait v2 was not impacted because it relies on vertical acceleration. Even after correction, the misalignment resulted in fewer steps being properly detected. Other metrics maintained higher agreement, as they were either aggregate measures (eg, gait speed) or based on estimates from a larger number of steps (eg, cadence).

**Table 6:** Test-retest ICC^a^ results between visits 1 and 2 in the ICANGO study for reference, comparison, and proposed algorithms (N=40).

Metric and walk style			PKMAS^b^, ICC (95% CI)		Gait v2, ICC (95% CI)		Gait v3, ICC (95% CI)
**Cadence (step/minute)**							
	Normal	0.85 (0.69-0.92)		0.82 (0.67-0.90)		0.84 (0.69-0.92)	
	Fast	0.86 (0.76-0.93)		0.85 (0.74-0.92)		0.86 (0.75-0.92)	
	Slow	0.84 (0.53-0.93)		0.58 (0.28-0.77)		0.83 (0.45-0.93)	
**Stride time (seconds)**							
	Normal	0.82 (0.65-0.91)		0.82 (0.67-0.90)		0.81 (0.64-0.90)	
	Fast	0.89 (0.80-0.94)		0.87 (0.77-0.93)		0.71 (0.52-0.84)	
	Slow	0.85 (0.57-0.93)		0.75 (0.43-0.88)		0.84 (0.55-0.93)	
**Stride length (m)**							
	Normal	0.91 (0.75-0.96)		0.86 (0.71-0.93)		0.91 (0.83-0.95)	
	Fast	0.85 (0.73-0.92)		0.87 (0.77-0.93)		0.85 (0.73-0.92)	
	Slow	0.80 (0.53-0.90)		0.78 (0.54-0.89)		0.81 (0.64-0.90)	
**Gait speed (m/s)**							
	Normal	0.81 (0.57-0.91)		0.84 (0.67-0.92)		0.84 (0.66-0.92)	
	Fast	0.78 (0.62-0.88)		0.79 (0.64-0.88)		0.78 (0.62-0.88)	
	Slow	0.83 (0.42-0.93)		0.77 (0.31-0.90)		0.80 (0.39-0.92)	

^a^ICC: intraclass correlation coefficient.

^b^PKMAS: ProtoKinetics Movement Analysis Software.

## Discussion

In this work, we presented a step-by-step description of the updates made to the SKDH [36] gait module, both within the algorithm subblocks and in the overall algorithm performance relative to a ground-truth GAITRite/PKMAS system. By applying the updated algorithm to healthy adult and pediatric populations in the ICANGO and MAGIC studies, and evaluating it across multiple comparison methods, these updates demonstrated significant improvements over the previous version of the gait module. This work introduces a generalizable, regulatory-grade framework for remote gait estimation using a single lumbar sensor—incorporating dynamic QC, robust axis-adaptive gait detection, and deployment-ready processing pipelines. These innovations move the field from algorithm development toward validated clinical deployment.

Estimation of the mean step time for a gait bout showed strong agreement, with higher Pearson correlation coefficients, ICCs, and RMCC. While per-stride step time is estimated as a final output of the gait algorithm, having an a priori estimate of the mean step time for the walking bout allows for dynamic adjustment of the gait event detection method as well as stride QC. This, in turn, minimizes the need for manual adjustment of algorithm parameters by the end user, which should support generalizability as the algorithm is deployed in patient populations. With these parameters—and those used in peak detection (eg, minimum peak prominence)—setting them based on the training set, whether visually or using ground-truth data, and then validating them in the test set provides confidence in their generalizability. However, future work might explore a sensitivity analysis of these parameters to assess their impact on the final results.

Accurate estimation of initial and final foot contact events is the critical foundational block for all subsequent gait metric calculations. We implemented a new approach for locating foot contacts, alongside implementations of several previously proposed methods for comparison [21,22,46-48,50]. Our method demonstrated superior *F*_1_-scores and MAE values compared with these methods across both IC and FC events. For IC events, the “g” (0.022 seconds) and “pe” (0.027 seconds) methods showed similar median MAE values; however, their *F*_1_-score performance was substantially worse (0.93 and 0.68, respectively) compared with the proposed method (MAE 0.023 seconds; *F*_1_-score of 0.98). FC results were even more favorable for the proposed method: only the “m” (gait v2) method achieved a comparable *F*_1_-score (median 0.98 vs 1.0), and none of the other methods approached its MAE performance (gait v3 median 0.018 seconds). A review paper compiling results from various IC/FC detection algorithms across multiple populations reported MAE values for IC events ranging from 0.013 to 0.148 seconds in healthy participants, and from 0.025 to 0.186 seconds in individuals with neurologic diseases, Parkinson disease, and hemiplegia [51]. Both the “g” and “pe” methods were among the best-performing algorithms in that review, aligning with the performance of existing methods observed in this work.

These improvements to the gait processing subblocks would, however, have limited value if they did not enhance the overall estimation of gait metrics. Across the 4 gait metrics presented, we observe consistently better results for the proposed gait v3 algorithm. Correlation values generally remain stable or show improvements, such as for cadence during slow and fast walks. ICC values further highlight these gains—particularly for spatial metrics, where stride length and gait speed ICCs increase from 0.50-0.74 in gait v2 to 0.81-0.90 across walking speeds. We also observe improvements in agreement for temporal metrics during slow and fast walking (eg, stride time ICC during slow walking increases from 0.75 in v2 to 1.00 in v3). These strengthened agreements are likely the result of both dynamic gait event detection and QC using the a priori estimation of mean step time, which eliminates the need to manually adjust parameters across different walking tasks. This should enable more reliable gait monitoring in at-home environments, where walking characteristics may vary depending on context. The improvements also result in a reduction of bias in gait metric estimation. These gains were observed and confirmed in the mixed-effects models used to evaluate performance across walking speeds and age groups. Specifically, the differences between gait v3 and the reference PKMAS values are consistently reduced or eliminated compared with gait v2, indicating reduced estimation bias across walking speeds. Moreover, estimation bias in younger age groups was also reduced, as shown in Table 5. Although some differences in gait speed and stride length remain in gait v3, these discrepancies are smaller.

The importance of the statistically significant improvements and bias reduction achieved by gait v3 becomes clearer when interpreted against meaningful change thresholds or minimal clinically important differences, which vary across pathological cohorts. Specifically, across walking speeds and age groups, gait v3 reduced bias in gait speed estimates by approximately 0.10-0.20 m/s compared with v2. This range aligns with the European Medical Agency’s qualification of the 95th percentile of gait speed, where changes of 0.10-0.20 m/s are considered clinically meaningful in Duchenne muscular dystrophy [56]. Similarly, in Parkinson disease, clinically important differences in gait speed have been reported up to ~0.20 m/s, depending on the assessment method [57]. The fact that the reductions in bias observed with gait v3 fall within these clinically meaningful ranges indicates that the updated algorithm enhances the ability to detect meaningful changes that gait v2 may have obscured. This improvement in accuracy is particularly important in clinical trial contexts, where misestimation at the level of clinically meaningful differences can inflate sample size requirements or mask therapeutic effects, and in real-world monitoring, where longitudinal changes must be confidently attributed to underlying clinical status rather than algorithmic error.

Beyond technical improvements, gait v3 also reflects a distinct design philosophy compared with conventional gait pipelines. Many existing approaches rely on multisensor fusion, subject-specific calibration, or data-driven modeling to optimize accuracy under controlled conditions—methods that can limit scalability and reduce compliance in real-world or decentralized settings. By contrast, gait v3 adopts a physically motivated, single-site lumbar design with embedded biomechanical constraints and dynamic QC, enabling calibration-free, interpretable, and robust performance across heterogeneous users and environments. This shift from calibration-dependent precision to calibration-free robustness emphasizes scalability, compliance, and longitudinal continuity—key requirements for home-based monitoring and decentralized clinical trials. By delivering reliable gait metrics from a single self-applied sensor, gait v3 reduces participant and site burden while maintaining regulatory-grade accuracy. Thus, its contribution extends beyond performance gains: it illustrates how biomechanically grounded, dynamically self-correcting algorithms can reconcile analytical rigor with real-world feasibility, advancing the broader adoption of digital gait end points in clinical research. In summary, gait v3 is well-suited to the emerging landscape of patient-centric, real-world digital health applications and decentralized clinical trials.

The most significant recent effort in gait algorithm design and validation was undertaken by the Mobilise-D collaboration, which recently published the technical validation of their gait platform in healthy adults as well as individuals with Parkinson disease, multiple sclerosis, chronic obstructive pulmonary disease, congestive heart failure, and proximal femur fracture. When evaluating multiple subblock algorithms, the best-performing method for IC detection demonstrated a sensitivity of 0.80 and absolute errors of 0.06 seconds in healthy participants, with similar performance across pathological cohorts [39]. Although the gait v2 algorithm implemented in SKDH has not been published in patient populations, the Mobilise-D results showed a wide range of ICC values for IC estimation (0.68-0.98) and step length (0.28-0.70) when validating the originally published algorithms on which gait v2 was based [39]. We report lower MAE (under 0.05 seconds) and higher *F*_1_-scores (over 0.95), although the values reported by Mobilise-D were obtained during simulated daily activities rather than prescripted tasks, which would likely contribute to the higher performance observed here. Following their gait subblock assessment and algorithm exploration, the Mobilise-D consortium also published full gait speed results based on the subblocks that performed best in their testing [38]. Within their full validation protocol—including simulated daily activities—algorithm performance during straight walking showed errors of –0.03 m/s (limits of agreement –0.18 to 0.11), with an absolute error of 0.07 m/s (limits of agreement 0.01 to 0.13) in healthy adults. This is very similar to our reported error of 0.024 m/s (95% CI –0.03 to 0.08) for normal straight-walk gait speed. Across all reported in-laboratory tasks, the Mobilise-D algorithm yielded an ICC of 0.74 (95% CI 0.66-0.81) for gait speed, slightly lower than our observed 0.82-0.90 across walking speeds, although the inclusion of simulated daily activities likely accounts for this difference. Overall, we report results that are comparable to, or better than, those from the Mobilise-D project, suggesting that SKDH may be a suitable alternative—pending further validation in pathological cohorts and in settings involving more simulated daily activities. This is particularly relevant for the dynamic thresholds used for QC checks. Although these thresholds were designed to be broad enough to encompass a wide range of physiological parameters, their underlying relationships may not always hold in pathological gait. Nevertheless, we maintain that dynamic, data-driven thresholds based on observed walking behavior are likely to outperform the original static thresholds in gait v2 when applied to pathological gait.

Assessing test-retest reliability across the 2 in-laboratory visits in the ICANGO study, ICC values were consistent for both the reference method and gait v3. This provides evidence to support its use as a reliable clinical outcome assessment and as a digital end point in clinical trials [44].

There are several limitations to this work. First, because the study population consisted solely of healthy individuals, we cannot infer the generalizability of the algorithm updates to patient populations. Although the inclusion of children and adolescents provides some indication of broader applicability, it remains insufficient to establish generalizability to clinical cohorts. Second, validation was restricted to prescripted in-laboratory walking tasks, even though multiple walking speeds were included. To address these limitations, future work will evaluate the algorithm’s validity in patient cohorts with distinct gait signatures, assess performance during simulated daily living, examine the impact of sensor misplacement, and compare results with other clinical outcome measures.

## Appendix

Multimedia Appendix 1 Supplementary gait results.

## Abbreviations

AP: anterior-posterior
CoM: center of mass
CWT: continuous wavelet transform
FC: final contact
FDA: Food and Drug Administration
IC: initial contact
ICANGO: In-Clinic and Natural Gait Observations
ICC: intraclass correlation coefficient
LMER: linear mixed-effects regression
MAE: mean absolute error
MAGIC: Monitoring Activity and Gait in Children
PKMAS: ProtoKinetics Movement Analysis Software
QC: quality control
RMCC: repeated measures correlation coefficient
SKDH: SciKit Digital Health

## Data Availability

Upon request, and subject to review, Pfizer will provide the data that support the findings of this study. Subject to certain criteria, conditions, and exceptions, Pfizer may also provide access to the related individual deidentified participant data. See [58] for more information.
