# Supplementary material for: Lumbar Acceleration Gait Estimation: “Step-by-Step” Algorithm Updates and Improvements
Source: J Med Internet Res. 2025 Dec 12;27:e72831. doi: 10.2196/72831 (PMC12743242; doi:10.2196/72831)
Supplement: Multimedia Appendix 1 [file jmir_v27i1e72831_app1.docx]

## Appendix: Supplementary Gait Results

Table 1 LMER results for IC and FC estimation for MAE and F1-scores, and contrasts between methods relative to gait v3.

|  | MAE | | | F1-score | | |
| --- | --- | --- | --- | --- | --- | --- |
| Method | LS Mean (95% CI) | Method - gait v3 (95% CI) | Diff. p-value | LS Mean (95% CI) | Method - gait v3 (95% CI) | Diff. p-value |
| *IC* |  |  |  |  |  |  |
| gait v3 | 0.026 (0.03 - 0.02) | - | - | 0.968 (1.00 - 0.94) | - | - |
| gait v2 | 0.074 (0.08 - 0.07) | 0.047 (0.04 - 0.05) | < 0.001 | 0.959 (0.99 - 0.93) | -0.009 (-0.06 - 0.04) | 0.57824 |
| g | 0.026 (0.03 - 0.02) | 0.000 (-0.01 - 0.01) | 0.88278 | 0.815 (0.84 - 0.79) | -0.153 (-0.20 - -0.10) | < 0.001 |
| b | 0.083 (0.09 - 0.08) | 0.057 (0.05 - 0.06) | < 0.001 | 0.891 (0.92 - 0.86) | -0.077 (-0.13 - -0.03) | < 0.001 |
| zcross | 0.043 (0.05 - 0.04) | 0.017 (0.01 - 0.02) | < 0.001 | 0.922 (0.95 - 0.89) | -0.046 (-0.09 - 0.00) | 0.00361 |
| zpeak | 0.110 (0.11 - 0.11) | 0.083 (0.08 - 0.09) | < 0.001 | 0.815 (0.84 - 0.79) | -0.153 (-0.20 - -0.10) | < 0.001 |
| pe | 0.033 (0.04 - 0.03) | 0.006 (0.00 - 0.01) | 0.00438 | 0.648 (0.68 - 0.62) | -0.320 (-0.37 - -0.27) | < 0.001 |
| sk | 0.078 (0.08 - 0.07) | 0.052 (0.05 - 0.06) | < 0.001 | 0.908 (0.94 - 0.88) | -0.060 (-0.11 - -0.01) | < 0.001 |
| *FC* |  |  |  |  |  |  |
| gait v3 | 0.023 (0.03 - 0.02) | - | - | 0.971 (1.01 - 0.94) | - | - |
| gait v2 | 0.065 (0.07 - 0.06) | 0.042 (0.04 - 0.05) | <0.001 | 0.892 (0.93 - 0.86) | -0.079 (-0.13 - -0.03) | <0.001 |
| g | 0.058 (0.06 - 0.05) | 0.035 (0.03 - 0.04) | <0.001 | 0.713 (0.75 - 0.68) | -0.258 (-0.31 - -0.21) | <0.001 |
| pe | 0.058 (0.06 - 0.05) | 0.035 (0.03 - 0.04) | <0.001 | 0.511 (0.55 - 0.48) | -0.460 (-0.51 - -0.41) | <0.001 |


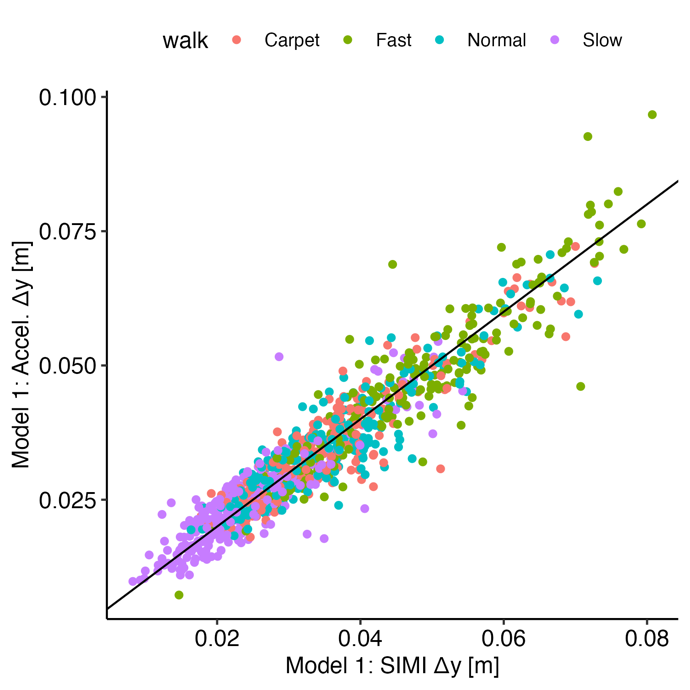

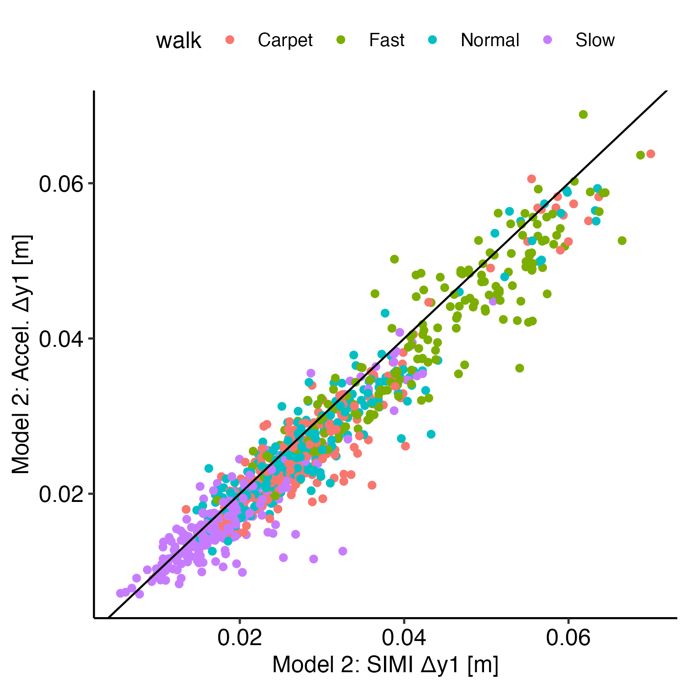


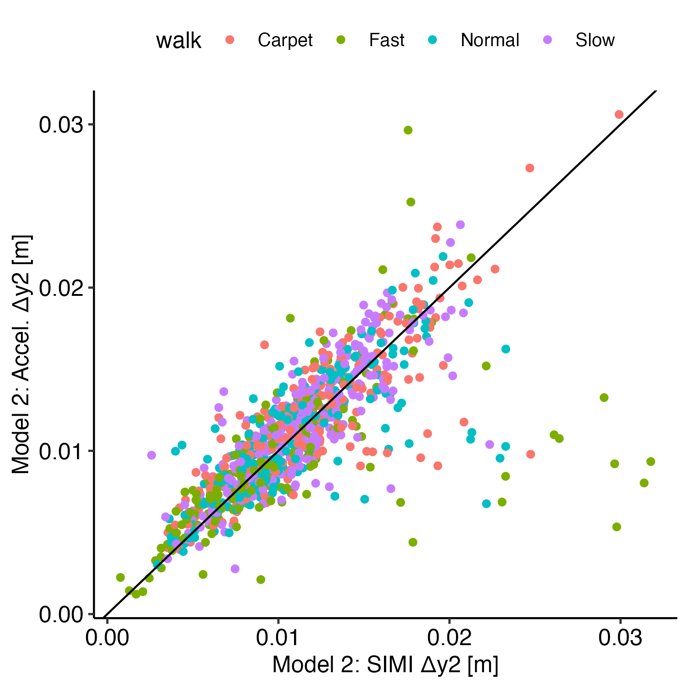


Figure 1 Inverted pendulum model center of mass change in height estimations. Uses initial and final contact events from PKMAS timings to isolate effect of estimation of center of mass height change.

Table 2 Step length agreement with reference for the two inverted pendulum models. Note that both contact events and center of mass change in height are taken from reference systems, isolating the effect of the pendulum model used. This also is why the values are quite different from those for the full gait algorithm stride length estimation in the main paper.

|  | Visit 1 Correlation | | All visits ICC | |
| --- | --- | --- | --- | --- |
| Walk | Model 1 | Model 2 | Model 1 | Model 2 |
| Carpet | 0.838 | 0.805 | 0.185  (-0.02, 0.553) | 0.594 (-0.05, 0.85) |
| Fast | 0.915 | 0.825 | 0.420  (-0.05, 0.78) | 0.685 (0.09, 0.87) |
| Normal | 0.855 | 0.805 | 0.351  (-0.04, 0.73) | 0.734 (0.20, 0.89) |
| Slow | 0.902 | 0.867 | 0.421 (-0.06, 0.78) | 0.842 (0.70, 0.92) |


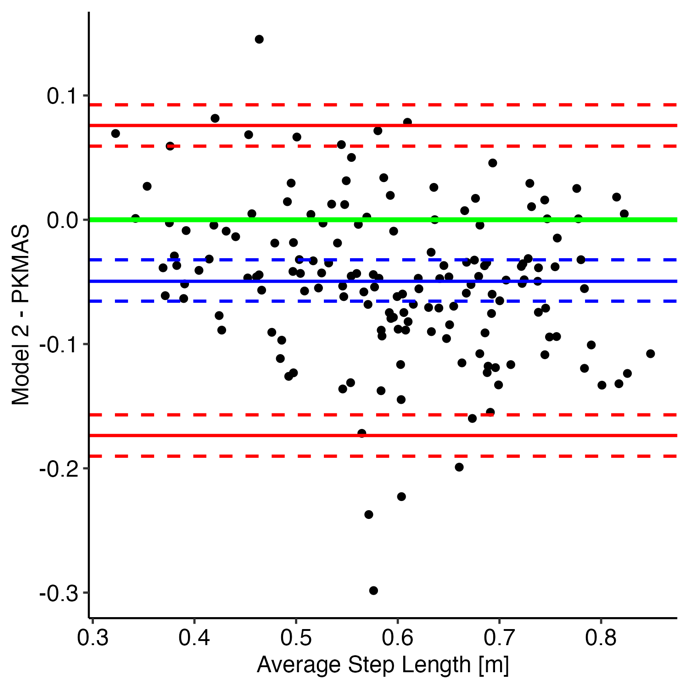

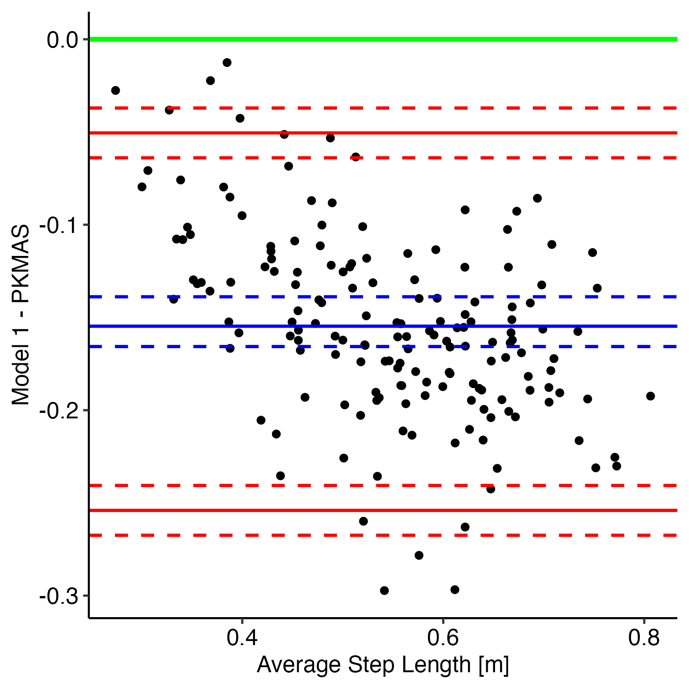


Figure 2 Estimated average step lengths over tasks using repeated measures from Visit 1, compared to reference. [Left] Inverted pendulum model 1 [Right] Inverted pendulum model 2.


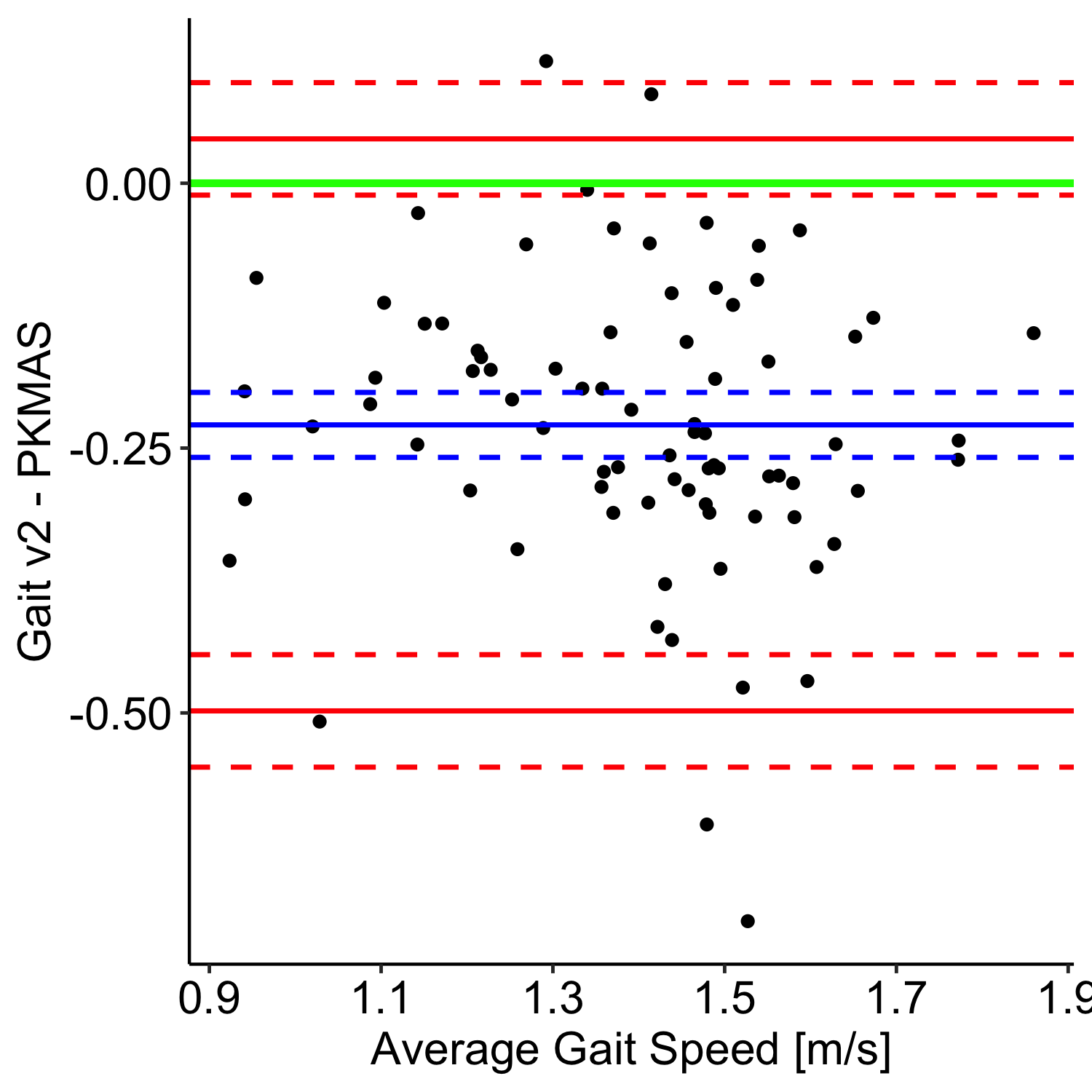

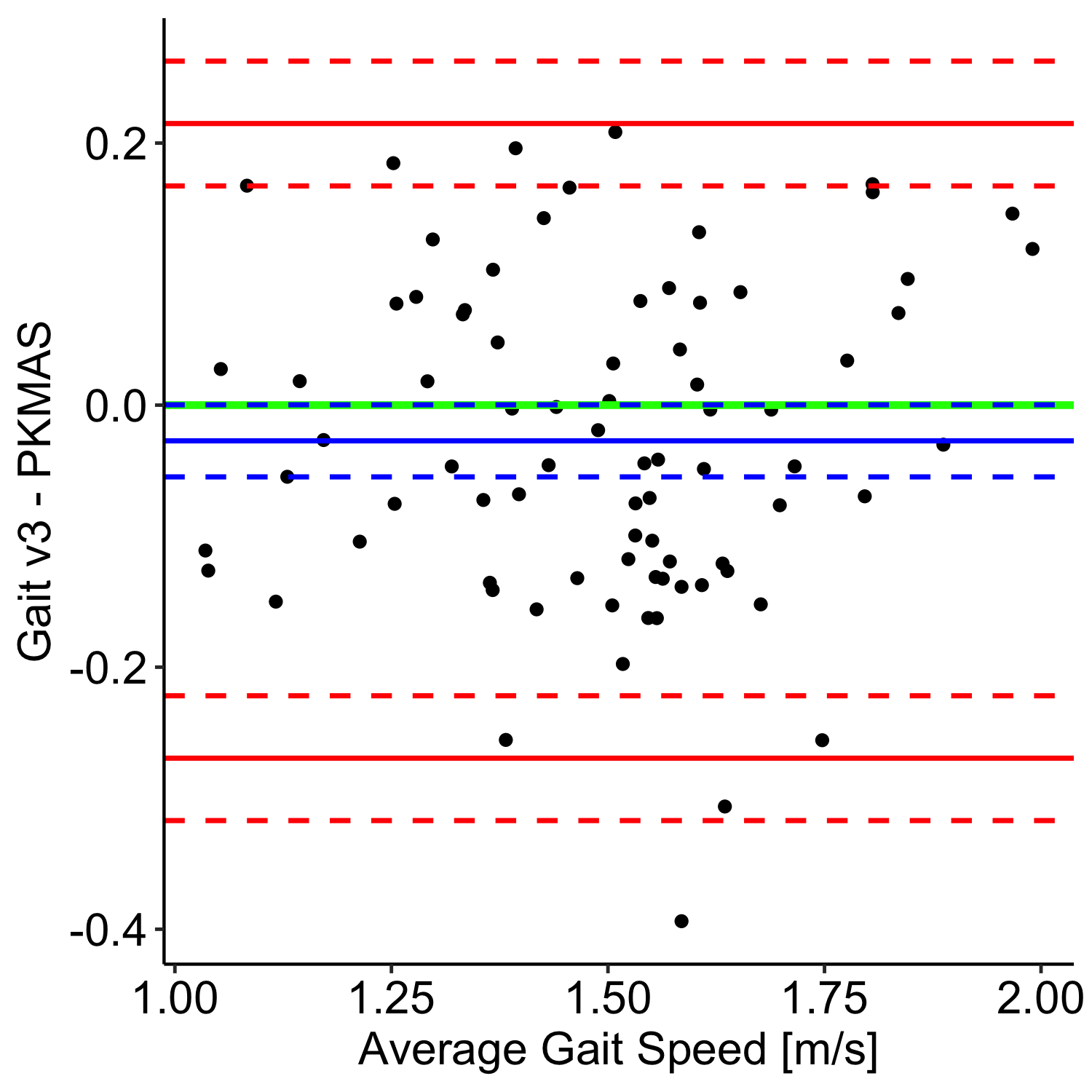


Gait v2, Fast walk Gait v3, Fast walk


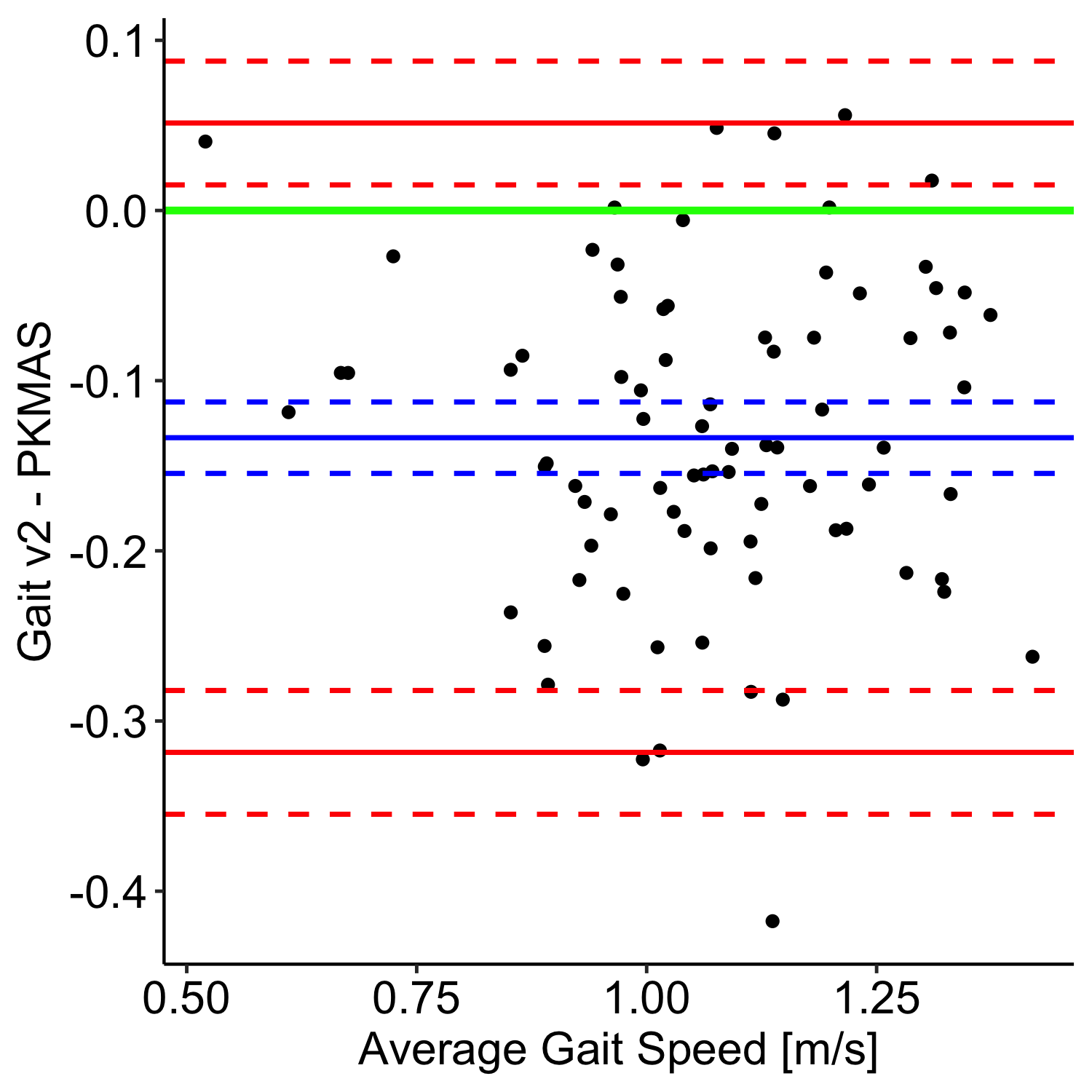

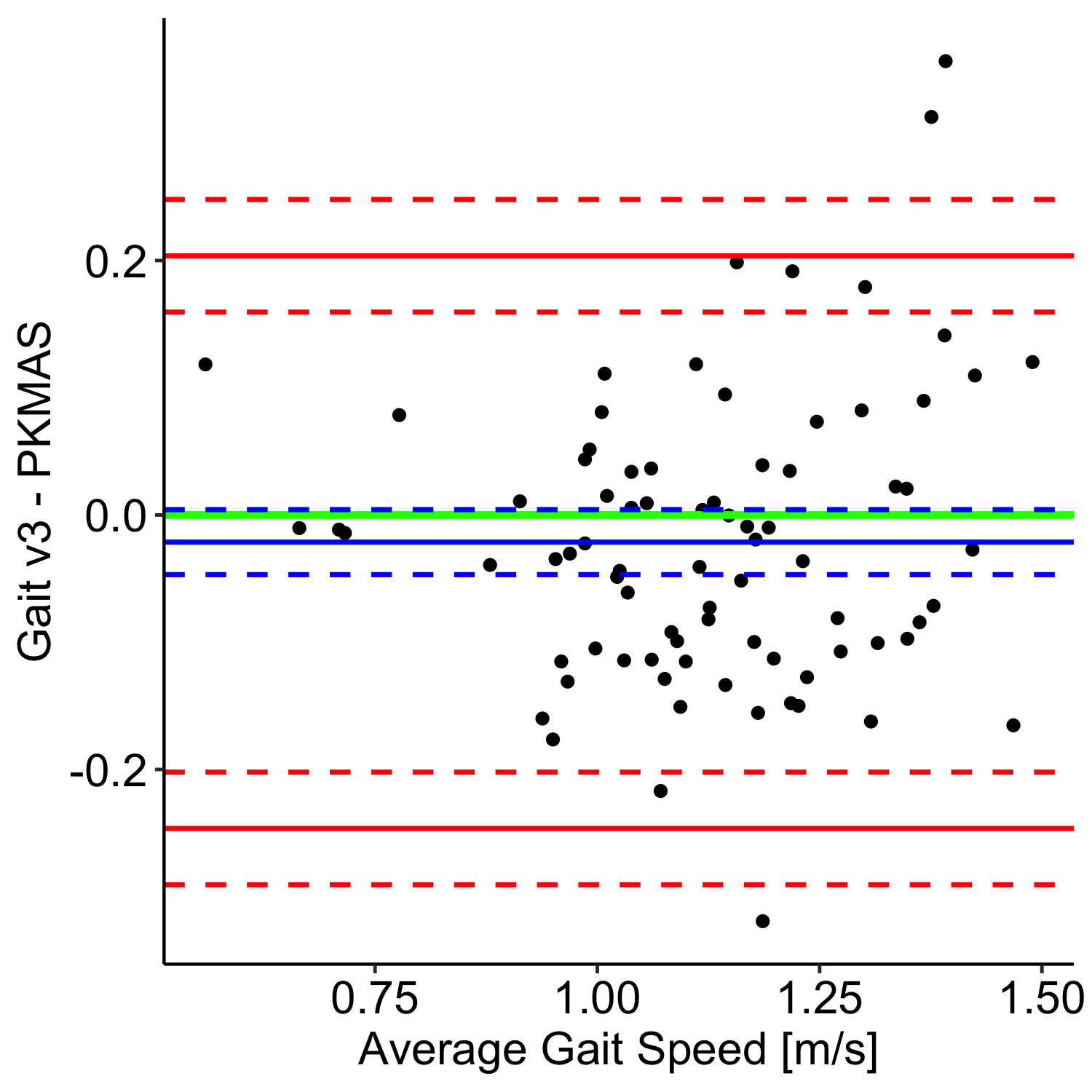


Gait v2, Normal walk Gait v3, Normal walk


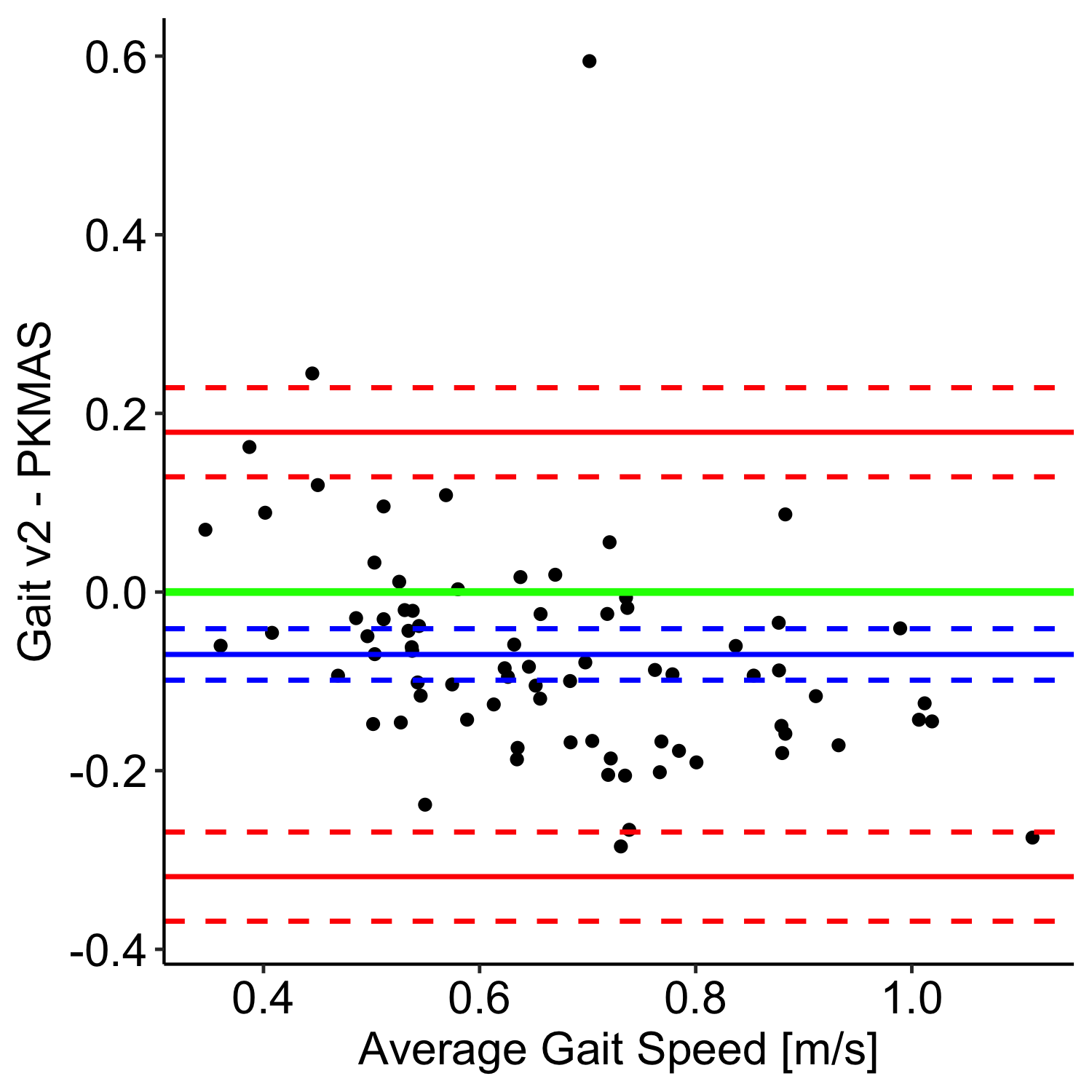

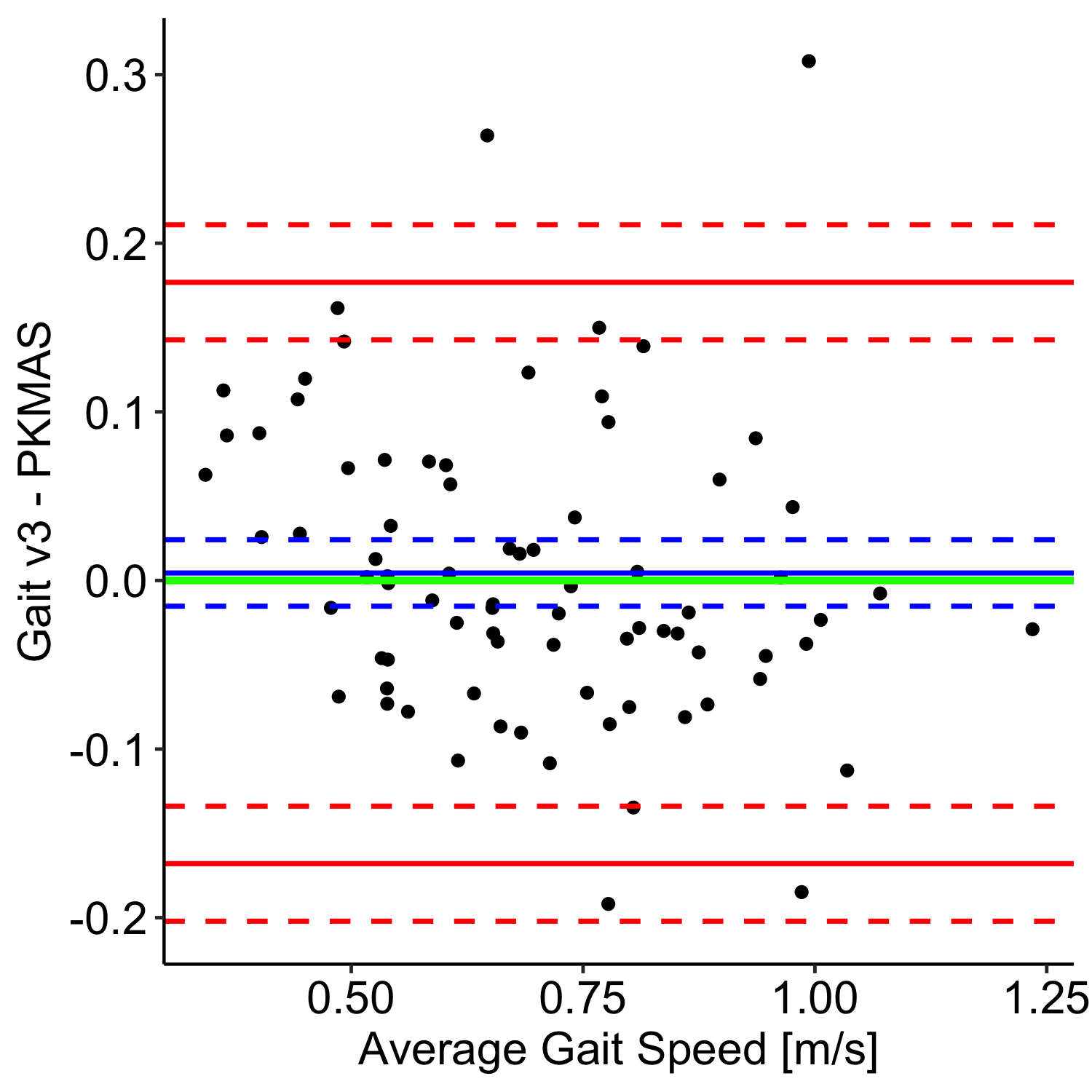


Gait v2, Slow walk Gait v3, Slow walk

Figure 3 Bland-Altman plots of gait speed results across walking speeds for visit-averaged data
